# Microplastics and Trash Cleaning and Harmonization (MaTCH): Semantic Data Ingestion and Harmonization Using Artificial Intelligence

**DOI:** 10.1021/acs.est.4c02406

**Published:** 2024-11-11

**Authors:** Hannah Hapich, Win Cowger, Andrew B. Gray

**Affiliations:** † University of California, Riverside, 900 University Avenue, Riverside, California 92521, United States; ‡ Moore Institute for Plastic Pollution Research, Long Beach, California 90803, United States

**Keywords:** plastic, microplastics, trash, natural language processing, harmonization, data management, artificial intelligence

## Abstract

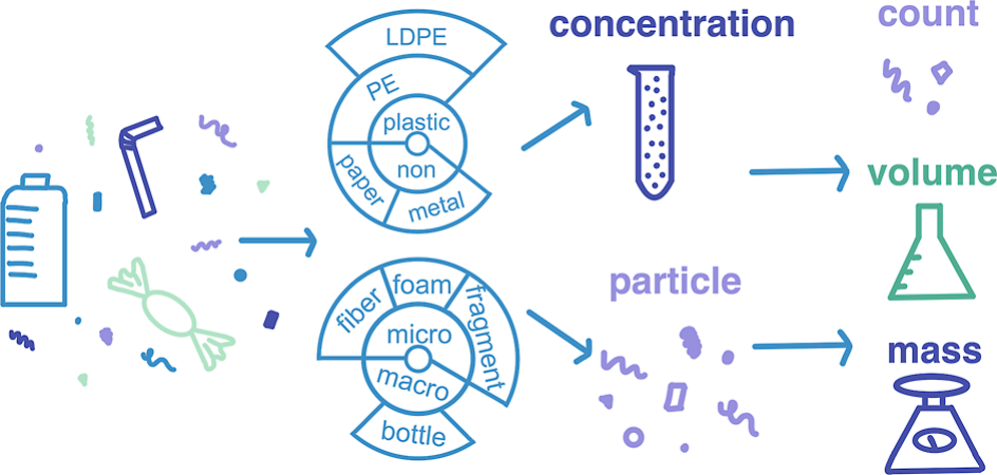

With the rapid expansion of microplastic research and reliance on semantic descriptors, there is an increasing need for plastic pollution data harmonization. Data standards have been developed but are seldom implemented across research sectors, geographic regions, environmental media, or size classes of plastic pollution. Harmonization of existing data is currently hindered by increasingly large datasets using thousands of different categorical variable descriptors, as well as various metrics used to describe particle abundance and differing size ranges studied across groups. For this study, we used manually developed relational databases to build an algorithm utilizing artificial intelligence capable of automatically curating harmonized, more usable datasets describing micro to macro plastic pollution in the environment. The study algorithm MaTCH (microplastics and trash cleaning and harmonization) can harmonize datasets with different formats, nomenclature, methods, and measured particle characteristics with an accuracy of 71–94% when matching semantically. All other non-semantic corrections are reported within a 95% confidence interval and with model uncertainty. All steps of the algorithm are integrated in an open-source software tool for the benefit of the scientific community and ease of integration for all plastic pollution data.

## Abstract 2

This study identifies semantic and non-semantic harmonization methods for microplastics and trash data and combines them into one unified algorithm, also available as an open-source web tool.

## 1 Introduction

Studies focused on trash (mismanaged waste > 5 mm in length^1^) and microplastic (plastics 1–5000 μm in length^1^) pollution have increased dramatically in number over recent years.^2^ Together, these studies have indicated that the majority of microplastics found in the environment are secondary products of degrading mismanaged plastic waste rather than primary emissions, pointing to a relationship in environmental occurrence between trash and microplastics.^3^ Microplastics and trash are diverse environmental pollutants that are difficult to query and quantify, as we generally describe them with incomparable categorical variables, and report environmental concentrations composed of varying reporting metrics and particle size ranges.^4^ Microplastics data is not currently standardized and is therefore less easily or reliably comparable between studies,^5,6^ leading to many calls for both standardization^6−9^ and harmonization.^5,10,11^ Nearly all propositions for standardization or harmonization have focused either on nano, micro,^5,9^ or macro^8,12^ particles, whose size domain thresholds are often arbitrary and inconsistent between groups.^13,14^ Given the intrinsic relationship between trash and microplastics in their environmental occurrence and categorical semantics, it follows that data management strategies for microplastics and trash should be harmonious.^15^

Standards have been developed for managing mandated trash assessment data^8^ and tabulating microplastics data with respect to specific reporting guidelines.^9^ Such strategies serve as hubs for accumulating already standardized data and are often specific to certain geographic regions, study media, or government protocols.^8,10,16^ Certain database structures may be better suited for data at the sample level (reported as a concentration) or the particle level (information reported for individual particles). Standardization is limited by the rate at which scientists, government organizations, nongovernmental organizations (NGOs), or industry adapt to such protocols. Additionally, most protocols do not have a strategy to utilize data that does not fit their standardized structures, which alienates potentially useful data. In these cases, users must perform data harmonization manually. It is particularly important in the field of microplastics monitoring to utilize existing databases due to the cost and time prohibitive nature of the field, wherein it commonly requires up to thousands of dollars and tens of hours to process a single sample, making data from each monitoring study highly valuable.

Harmonization posthoc methods, wherein the harmonization of data occurs after a study is complete and no prior consideration for data standards is necessary, allow the inclusion of more data but are limited by the ease at which foreign data can be assimilated to a common structure.^12^ Manual harmonization can take weeks for even a single dataset depending on its size, again leading to underutilization of plastics databases.^2^ Previous work on harmonizing trash data has focused on manually extracting categorical variables to develop broad encompassing data structures, including databases such as the Trash Taxonomy, wherein terms used to describe trash characteristics are detailed in a relational table database.^12^ However, there exists no approach that automatically harmonizes macro debris and microplastics data from nonspecified formats or with unknown categorical descriptors.^2^ Best practices for the development of database structures have remained a manual undertaking that should be performed with the input of a wide array of stakeholders, though the addition of new terms is prohibitively effort-intensive. An automated approach to data harmonization would allow for quick ingestion of data from new studies, leading to larger, more valuable databases.

The field of microplastics and trash is not the first to encounter such issues. Many divisions of the environmental and biological sciences have similar problems, which will worsen over time with ever-growing datasets and a focus on curating “big data” to identify knowledge gaps and answer key questions.^17^ Previous work has assessed the use of natural language processing (NLP) algorithms as a means for information retrieval to assemble databases and organize their taxonomic structures.^18,19^ Until recently, the technology available consisted of different pattern matching and syntactic/semantic parsing, some of which rely on extracting exact matches, and most have a narrow application range tailored to a specific subfield.^19^ Results from early exploration of NLP for scientific data curation were discouraging^20^ and may have led to underutilization.

NLP technology has vastly improved in accuracy and efficiency just over the past few years, primarily a result of increases in computing power and the development of open-source artificial intelligence (AI) software capable of employing transformers and embeddings.^21^ Transformers are a type of neural network structure able to interpret data nonsequentially.^22^ The first step in using a transformer is to encode words as vectors, of which embeddings are one of the most efficient vector types to compare and derive meaning. Advanced open-source language models, such as BERT (bidirectional encoder representations from transformers)^21^ and GPT (generative pre-training transformer) (provided through OpenAI),^23^ have made it possible for scientists to now utilize the power of embeddings and AI to rapidly automate harmonization of categorical data—including those for trash and microplastics.

In addition to semantic harmonization—referring to combining categorical variables based on their semantic meaning in addition to their structural similarity—effective and transferrable microplastics data reporting is challenged by the incongruence of concentration data spanning different particle size ranges and a paucity of the particle-level reporting required to minimize error and ensure accurate representation. Microplastic sample concentration is most often reported on a particle count basis and is dependent on the size range of microplastics characterized, as microplastic occurrence data show particle counts generally increase with decreasing size in the form of an inverse power law relationship.^24−27^ Inconsistencies may also arise from differing reported dimensions (i.e., length, mass, projected surface area, and volume).^26^ Our framework includes non-semantic harmonization methods that both rescale studied-size ranges and convert between count, volume, and mass given basic particle characteristics. Our framework also attempts to improve the accuracy and transparency of such non-semantic harmonization methods by introducing a large database of polymer densities (*n* > 77,000) and reporting 95% confidence intervals and model error.

This study aims to (1) create a data management workflow to merge terminologies for both trash and microplastics in one unified querying system, (2) integrate an automated NLP step into the workflow to account for foreign terminology, (3) create an automated strategy for rescaling heterogeneous microplastic concentrations and particle metrics, (4) validate the performance of the automated data harmonization pipelines relative to manual curation, (5) generate open-source web tools for scientists to rapidly leverage the created algorithm, and (6) create a generalizable approach to perform such analyses so they may be applied in other fields. Through the use of NLP and newly assessed data structures, we aim to ensure that all descriptors of these complex pollutants are retained during harmonization to provide datasets that are comparable between studies without losing any of the rich descriptive information provided to better support plastic pollution investigation and future management.

## 2 Materials and Methods

### 2.1 Model Development: Semantic Data Harmonization

#### 2.1.1 Microplastics and Trash Taxonomy

To assess the current lexicon of microplastics researchers in describing particles, we used Google Scholar to search for studies related to microplastic occurrence in drinking water (75% tap and 25% bottled) and an environmental compartment of interest—rivers—including multiple media types with a focus on surface water (95% surface water, 4% sediment, and 1% river discharged effluent). In total, we reviewed 57 studies totaling 1186 samples (Table S1), recording concentration data and a comprehensive list of all unique terms used to describe particles, all of which included some instrumental verification that particles found were plastic. Data was manually extracted from article text and reported in the format described in SI file “User Guide”, Section 3 “Data Structuring”. None of the reviewed studies (Table S1) reported individual particle data; therefore, we focused on harmonizing sample level data for our review.

Our goal was to build on the existing Trash Taxonomy to integrate terms describing both microplastics and macro debris into one querying system.^12^ We created alias tables that first relate any synonymous terms (e.g., "LDPE" and“low density polyethylene”), as well as hierarchical tables to relate child and parent terms [e.g., “PE” (polyethylene) is the parent term to both “LDPE” (low-density polyethylene) and “HDPE” (high-density polyethylene)] (Figure S1). Through conducting this review, the primary reported categorical characteristics were the morphology (shape), material, and color. All types of categorical variables have their own alias tables, and hierarchical tables were generated for material and morphology. Similarity between terms used to describe morphology and material is calculated via eq 1
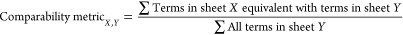
1Equation 1: Comparability metric to be computed for material or morphology between two sheets (sheet *X* and sheet *Y*), where the numerator describes the number of terms present in both sheet *X* and sheet *Y*, and the denominator describes the total number of terms in sheet *Y*.

Microplastic morphologies can be easily differentiated from trash, as they all have the parent term “microplastic” in the morphology hierarchy table. To differentiate materials, specific polymer names to describe microplastics are all under the parent term “plastic” in the materials hierarchy table. These merged microplastic and trash taxa allow all plastic pollution data to be studied contemporaneously, regardless of size.

#### 2.1.2 Embeddings and Querying through AI

We used the text-embedding-ada-002 embedding model and API from OpenAI^28^ to generate our embeddings—the vector described earlier needed for relating terms via NLP, with each term having a corresponding vector embedding—deployed in R (4.3.0) and RStudio (23.09.0). This model was chosen on the basis of accuracy, speed, and cost. Accuracy was assessed by attempting to reproduce our alias relational tables through matching aliases to their associated prime terms by the similarity of their associated embeddings.

The alias tables consist of one “prime” term that acts as the key linking to other tables in the relational database and other synonymous terms as “aliases”. Embeddings were generated for 1326 morphology aliases and 609 material aliases. Of these, 441 morphologies and 406 materials were designated to be “prime” terms, and 885 morphologies and 203 materials were designated as synonymous “alias” terms. Using the “chRoma” package^29^ that streamlines vector database management, we queried the top five matches as determined via dot product between each embedding vector (see data availability statement to access open-source code). We compared these results to the actual prime term for that alias in the alias tables—as outlined in Section 2.1.1—and reported those that were correctly matched to their prime term, as well as those that contained the correct prime term in the top 5 matches as ranked by percent similarity. We also used a database not previously integrated from an urban litter study^30^ and compared embedding matches to manual matches.

### 2.2 Model Development: Non-Semantic Data Harmonization

#### 2.2.1 Particle Count to Mass Conversion

For the sake of simplicity, when trying to traverse between count to mass-based concentration estimates, some have assumed a constant density and spherical volume, which greatly increases error margins.^31^ Kooi and Koelmans (2019)^26^ identified the problem of applying subjective morphological and polymeric descriptors to microplastic particles and rather proposed reporting as continuous distributions. In their study, ranges were established for the *L:W:H* (length to width to height) ratios of five common morphology types (fiber, fragment, film, foam, and sphere).^26^ We have integrated these proposed *L:W:H* ratios into our algorithm to convert morphology and length information into particle volume. Error was calculated by deriving a 95% confidence interval for each axis given the reported possible ranges, assuming a normal distribution. A measurement error of ±5% for particle length was also included to better characterize all possible sources of uncertainty.

**Figure 1:**
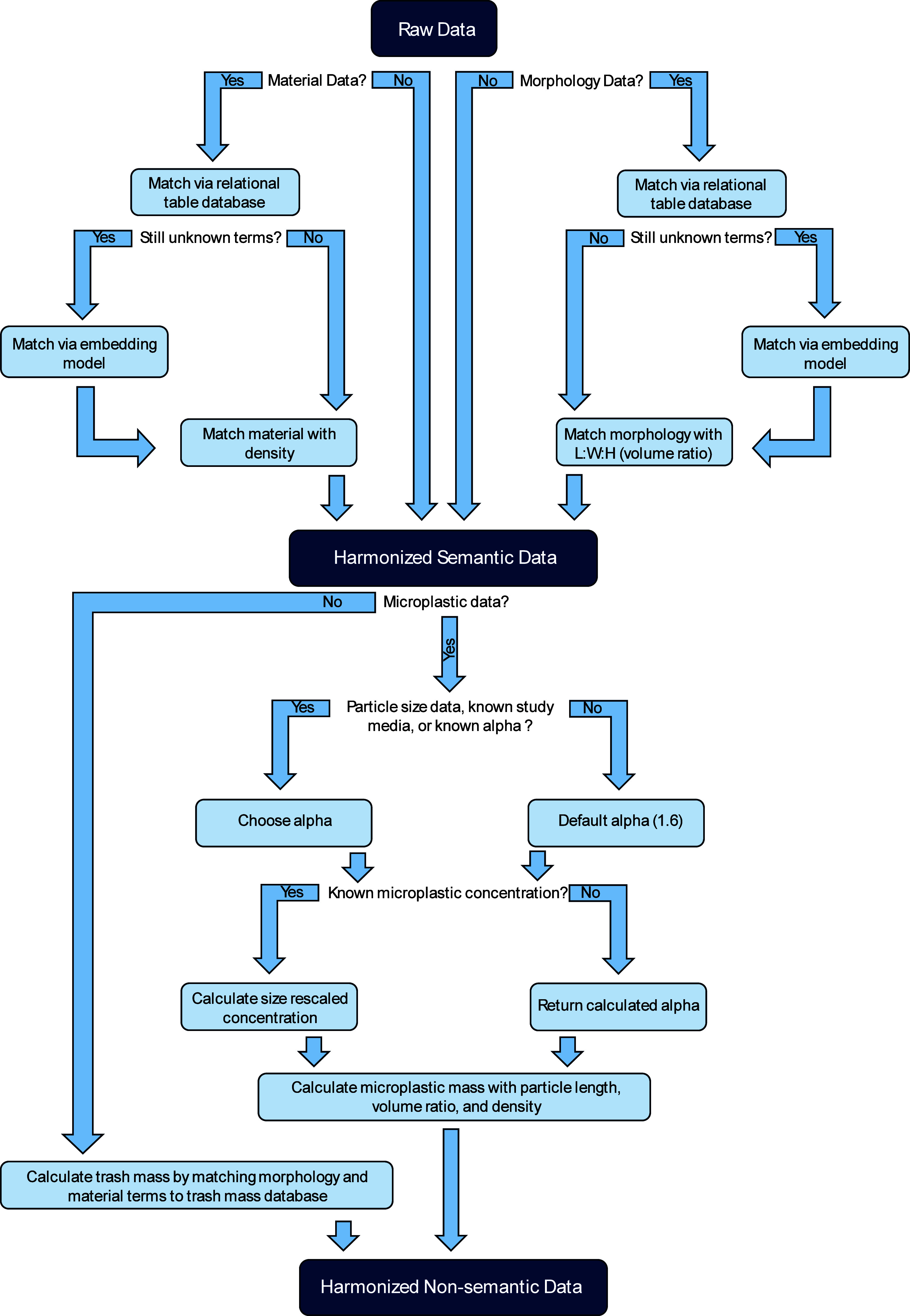
Microplastics and trash cleaning and harmonization (MaTCH) workflow schematic. Dark blue boxes represent data inputs and outputs. Light blue boxes represent model operations. Text outside of boxes and medium blue arrows represent decision trees.

To obtain mass, we then multiply the volume estimates, as outlined above, by material density. To obtain density, we curated a database including over 77,000 polymer density measurements (provided by Dale Kipp of MatWeb),^32^ for which we computed median values,^33^ adding to existing databases^26,34^ of *n* = ∼ 1000 polymer densities. If only a range of densities were provided, a Gaussian distribution was assumed, and a 95% confidence interval was derived. If individual density measurements were available, as in the case of our internally curated dataset, confidence intervals were derived using actual values. If no range or individual measurements were provided, then the average coefficient of variation of polymers with known error was applied. Our updated database contains 310 unique densities that are specific constituents of 43 parent polymer classes. By using densities derived from a larger number of measurements, we hope to better encompass the possible ranges of actual plastic particle densities while better representing uncertainty (Figure 1). In order to estimate mass for macro debris, we have used the method outlined in Cowger et al. (2022), which includes a literature review of studies that report litter masses to obtain average values for 205 distinct trash morphologies (Figure 1).^30^

If only sample level data is provided, we simulate particle data according to the morphological/material proportions provided and then follow the workflow mentioned above. Additionally, although we expect a majority of user input terms to be known within our database, the use of embedding models to describe polymer or morphology could lead to compounding error when calculating mass if improper matching was to arise for morphology or material.

#### 2.2.2 Particle Size Rescaling

Investigations of continuous distributions of microplastic sizes have found that smaller microplastics contribute a much larger proportion to total concentrations by count.^26,34^ Kooi et al. (2021) derived several inverse power law models^26^ to describe microplastic size distributions for various environmental media.^34^ In their approach, a meta-analysis was performed on studies that report microplastic size and relative abundance to develop alpha values (power law exponents) for probability density functions describing size. In the current study, we applied this method to any dataset with more than 5 size bins (the recommended minimum n by Nor et al., 2021^35^) to derive an alpha value (eq S1) (Figure 1).^26^ This value is used to calculate a correction factor (eq S2),^27^ which is then multiplied by the given concentration to describe the corrected range, which may be larger or smaller than the original value depending on if you want to expand or collapse your size range (Figure 1). Additionally, we integrated the standard error from the inverse power law model into our total error budget. Note, particle size rescaling is contingent only upon sample size information and not on any particle characteristic distributions, meaning there will be no compounding error derived from embedding match uncertainty. Additionally, note that rescaling is not applicable to macro debris (Figure 1). Degradation pathways suspected to be the cause for observed microplastic particle size distributions (PSDs) would not apply to macro debris that is typically a result of primary production, not secondary degradation.

### 2.3 Model Testing: Drinking Water vs Riverine Microplastic Occurrence Meta-Analysis Harmonization

To assess the applicability of our harmonization model to real world plastic pollution data, we merged curated datasets of microplastic occurrence in drinking water (*n* = 600) and in rivers across the United States (*n* = 586) (Table S1). These sources were chosen due to their suspected incompatibility in terms of particle size and concentration, with drinking water studies assumed to have lower concentrations and smaller particles observed. Our goals were to assess differences in semantics and our ability to make cross-study comparisons more concise as well as rescaling concentrations to analyze subsequent transformations in both occurrence and semantics.

To rescale the concentrations, correction factors were obtained for each sample. Values from Kooi et al. (2021) were used for studies in freshwater surface, freshwater sediment, and effluent discharged into streams (α = 2.64 ± 0.01, 3.25 ± 0.19, and 2.54 ± 0.04, respectively) (Figure 1). An alpha value for drinking water was developed (α = 1.64 ± 0.55), in which 11 samples were isolated that met the criteria of having ≥5 reported-size bins. The method described in Section 2.2.2 above was used to obtain a corrected concentration that describes the full 1–5000 μm range.^34^

When semantic data was provided, morphology and polymer were converted to one of the prime terms in the database presented in this study (Figure 1). Correcting various samples with differing size ranges, as we have done in our meta-analysis, will also have implications on the final proportions of categorical variables, as each study has a unique correction factor. Results displaying the reported and corrected concentrations, morphologies, and materials are discussed below. While this case study utilizes our model’s non-semantic capability to correct for differing size ranges, see the Supporting Information section titled “Model Testing: Micro vs. Macro Roadway Debris -Meta-Analysis Harmonization” for a similar case study of count to mass conversions for different sized roadway debris.

### 2.4 Open-Source Web-Tools

We developed a simple user interface—the Microplastics and Trash Cleaning and Harmonization (MaTCH) app—that will perform all possible harmonization with a single upload is available at https://hannahhapich.shinyapps.io/match/. MaTCH will analyze the column headings in the uploaded data table to determine the format (particle or concentration) and which data cleaning operations can be performed (semantic matching, count to mass conversion, and size rescaling). Particle and concentration test data are available for download to illustrate MaTCH’s features. This app was developed with the shiny,^36^ tidyr,^37^ skimr,^38^ chRoma,^29^ tibble,^39^ dplyr,^40^ data.table,^41^ data.tree,^42^ plotly,^43^ bs4 Dash,^44^ classInt,^45^ aws.s3,^46^ digest,^47^ DT,^48^ shinyTree,^49^ shinyhelper,^50^ and shinyWidgets^51^ packages in R (4.3.0) and RStudio (23.09.0).

#### 2.4.1 Semantic Merging Interface

If material and morphology are provided, a match through our relational table system is first performed (Section 2.1.1), and if the term is unknown, an embedding match is performed (Section 2.1.2). The algorithm defaults to the top embedding match, but also provides a dropdown list with the top five matches that allows the user to manually override this selection. With user approval, reported particle characteristic frequencies are saved to an Amazon S3 Cloud Database to help inform future algorithm development by indicating most commonly found particle types. Additionally, the tool provides rapid data visualization in the form of sunburst plots (hierarchical pie charts) for materials and morphologies and is available for download.

#### 2.4.2 Non-Semantic Merging Interface

The count to mass conversion tool allows users to upload data that must include at least length, morphology, and material. Additional variables can be included as outlined in the data template tab on the tool; however, they are not required and therefore do not need to be a complete column, as in the case of the “Sample Test Data” available on the homepage. Data may be uploaded in the form of particle data with actual values for each or as concentration data with particle size and proportions for morphology and material. Using the model and error calculations detailed above (Section 2.2.1), the output consists of volume, density, mass, and confidence intervals for each.

We also developed a tool for concentration rescaling that allows users to upload microplastic concentrations of their sample(s), studied size range, and desired extrapolated size range (within the 1–5000 μm microplastics size limit) to obtain rescaled concentrations. Users have the option to upload concentration data or particle level data. If binned concentration data with ≥5 bins or particle data are available, an alpha value will be generated for the sample calculated in accordance with methods detailed above (Section 2.2.2). If no study media or <5 size bins are provided, a default of α = 1.6 ± 0.5 will be used.^26^ These alpha values are used to derive a correction factor and corrected concentration for accompanying sample data.

## 3 Results and Discussion

### 3.1 Model Development: Semantic Data Harmonization

Of the 57 microplastic studies reviewed, only 13 reported their findings for categorical descriptors of microplastics. While several studies reported microplastic colors present in their samples, only three studies in our analysis reported proportion values, and color was therefore omitted from our comparability analysis. In total, this allowed for 156 comparability metrics to be derived analyzing the comparability of microplastic morphology and material (Figure S2).

Using the comparability metric (eq 1), we found mean comparability between unharmonized studies was 34.0% for morphologies and 6.4% for materials. The number of 100% comparable observations was 31 for morphologies and 0 for materials. Alternatively, the number of 0% comparable studies was 80 for morphologies and 136 for materials. Our findings illustrate both a lack in reporting characteristic distributions among microplastic studies and little harmonization between groups that do report.

Through the validation of the MaTCH workflow, we found that 70.62% of morphologies and 76.14% of materials were a top match to their correct aliases (Figure S2). Additionally, 87.91% of morphologies and 94.32% of materials had a correct match in the top 5 matches (Figure S2). We also assessed terms outside of our database obtained from a litter accumulation study on urban roadways in Southern California^30^ and a microdebris roadway study^52^ to further verify accuracy. Of the total 204 descriptors used between these studies, 119 terms were unknown to our database, of which 93 (78%) were correctly matched to a prime term as compared to the manually harmonized dataset. Note that the results here reflect those obtained from running the analysis at the time of publication. The OpenAI embedding model used here is subject to change over time, whether it be due to actual changes in the model or intentionally injected randomness, so exact percentages may vary slightly over time. Also, note that the accuracy percentages reported above assume all terms must be matched via embeddings. Terms are first matched via relational table, and it is our expectation that this database—with thousands of terms—will be able to match user input descriptors a majority of the time, resulting in 100% accuracy for those terms; the use of the embedding model acts more as a secondary failsafe method.

However, there are still some limitations to embedding-based semantic matching strategies. When attempting to match full polymer names to common abbreviations (e.g., “polyethylene” and “PE”), our matches were much lower (38.56–47.32%). In embedding space, unabbreviated polymer names were more closely related to each other than to their corresponding abbreviations. For example, “polyethylene” would be closer to “poly(ethylene glycol)” than to its associated abbreviation “PE”. Conversely, “PET” (polyethylene terephthalate) would be more closely related to “PE” than to “polyethylene terephthalate”. To account for this technical limitation, known polymer abbreviations were excluded from our embedding database, though they are still included in the relational tables. This means incoming unknown terms will be matched to an unabbreviated alias first (e.g., “poly(ethylene)” would match to “polyethylene”) and then matches to common abbreviations are done via the relational tables (e.g., to “PE”).

We have developed a data validation routine that allows users in any field to assess the accuracy of embedding matches for their chosen field, available via our GitHub page. If user desired accuracy is achieved, our algorithm can be applied to any use case by swapping out relational tables. Also, note that our model has not undergone any fine-tuning (model training of user data) to better characterize our use case. With proper funding and sufficient training data, the accuracy of embedding matches will improve through fine-tuning.

Supported by the high accuracy found in our study, integration of embedding-based NLP and other AI technologies to match synonymous semantic terminology appears promising. These results illustrate the power of NLP to increase the automation of categorical data harmonization. Automated curation of big datasets will increase the usability of existing data and may expedite future research studies that rely on the synthesis of microplastics data. With low-to-no-cost models becoming more accessible, we believe future studies should consider these methods of data integration in the field of plastic pollution research and beyond.

### 3.2 Model Testing: Drinking Water vs Riverine Microplastic Occurrence Meta-Analysis Harmonization

**Figure 2:**
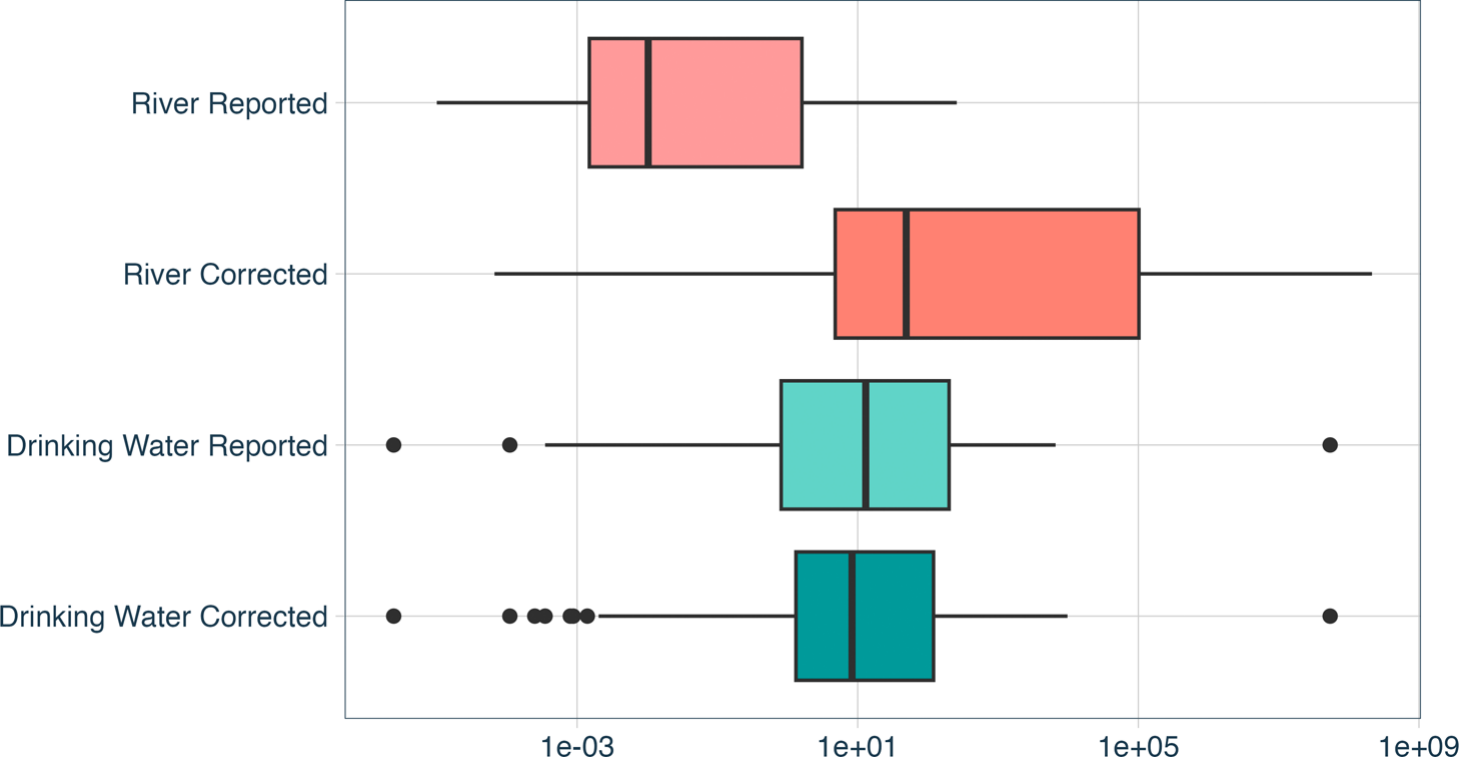
Microplastic concentrations (particle/L) before and after particle size rescaling. Data sourced from meta-analyses of the present study (Table S1) including data from microplastics monitoring of riverine systems (*n* = 586) and drinking water (*n* = 600).

Median concentrations of all drinking water studies curated were found to be 9.0 particles/L, which when run through MaTCH and corrected to the full microplastic particle size range (1–5000 μm) decreased to 6.03 ± 2.19 particles/L (Figure 2). Some drinking water studies included size ranges beneath 1 μm (filters with 0.2 μm pore size and no reported lower limit), resulting in a slight decrease in concentration when fitting to the 1–5000 μm distribution. Median river concentrations were 0.004 particles/L, increasing 4 orders of magnitude to 23.13 ± 1.53 particles/L when run through MaTCH (Figure 2). In contrast to drinking water, riverine studies focused on larger size ranges (median studied range of 355–5000 μm), likely due to analytical hurdles when dealing with environmental media. This resulted in a dramatic increase in concentration when size ranges were rescaled to the expanded 1–5000 μm range. This shift in riverine concentrations reversed the plotted interpretation of which media have a higher concentration. Though differences in analyzed size ranges were simply a result of differing objectives and analytical limitations across studies, when trying to draw a broad conclusion about particle abundance in different environmental compartments, uncertainties and errors can hide between microplastics datasets that are misaligned with one another.

**Figure 3:**
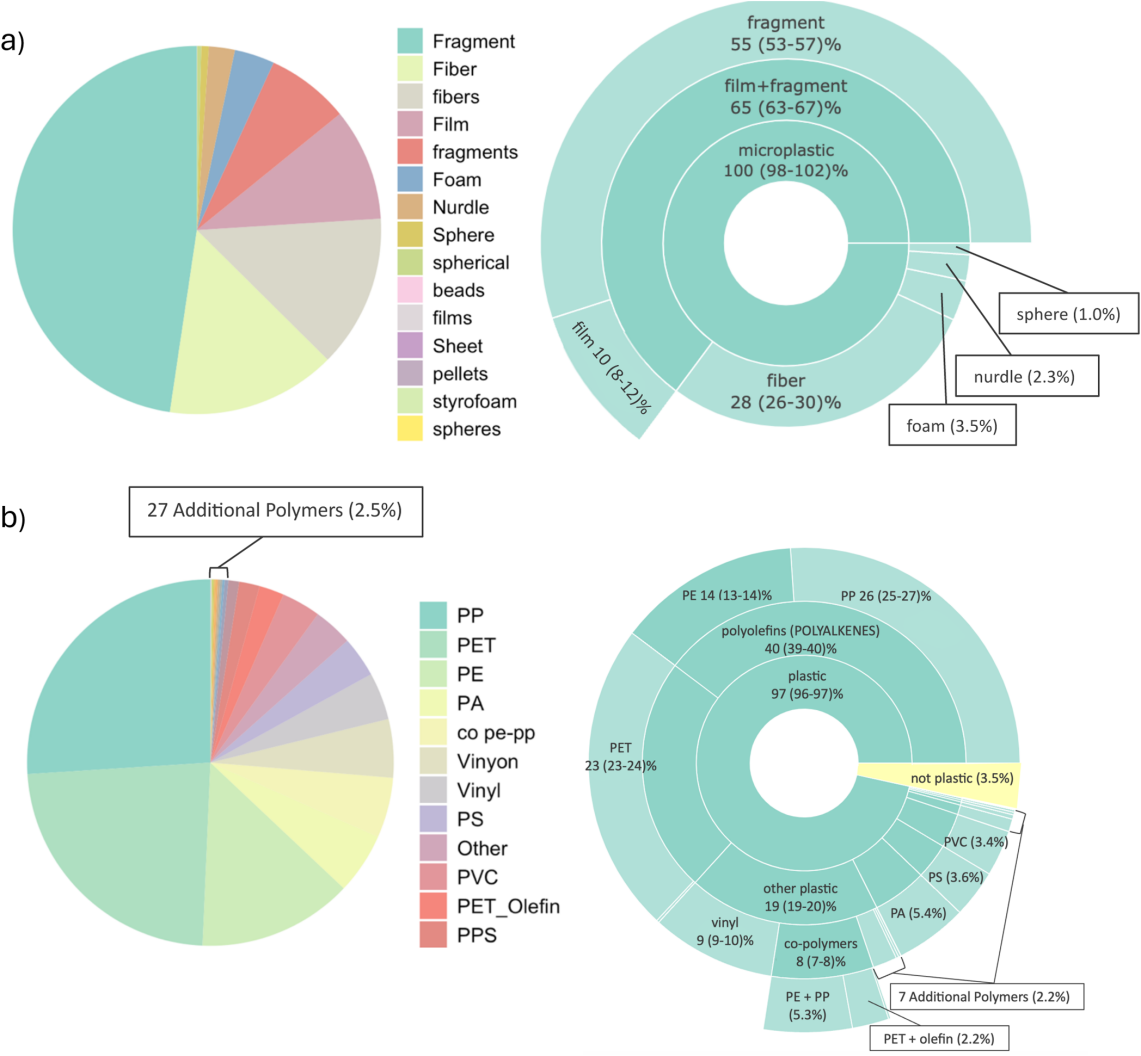
Change in morphological (a) and polymeric (b) categorical variables before and after semantic alignment. Illustration of hierarchical lumping via sunburst plot with 95% confidence intervals. (PP: polypropylene, PET: polyethylene terephthalate, PE: polyethylene, PA: polyamide, PS: polystyrene, PVC: polyvinyl chloride, and PPS: polyphenylene sulfide).

For studies reporting on morphology or polymer composition of their samples, categorical terms were run through MaTCH. Terms used to describe morphology were reduced from 15 to 6, and polymer terms were reduced from 39 to 25. Parent hierarchical terms were plotted in sunburst plots whether or not parent terms were explicitly used in sample data to help visualize all scales of classification simultaneously (Figure 3a,b). Using hierarchical structuring maximizes characteristic data comparability between studies while still retaining more detailed information from studies that use more specific terminology to better inform non-semantic harmonization techniques (Figures 1 and 3).

**Figure 4:**
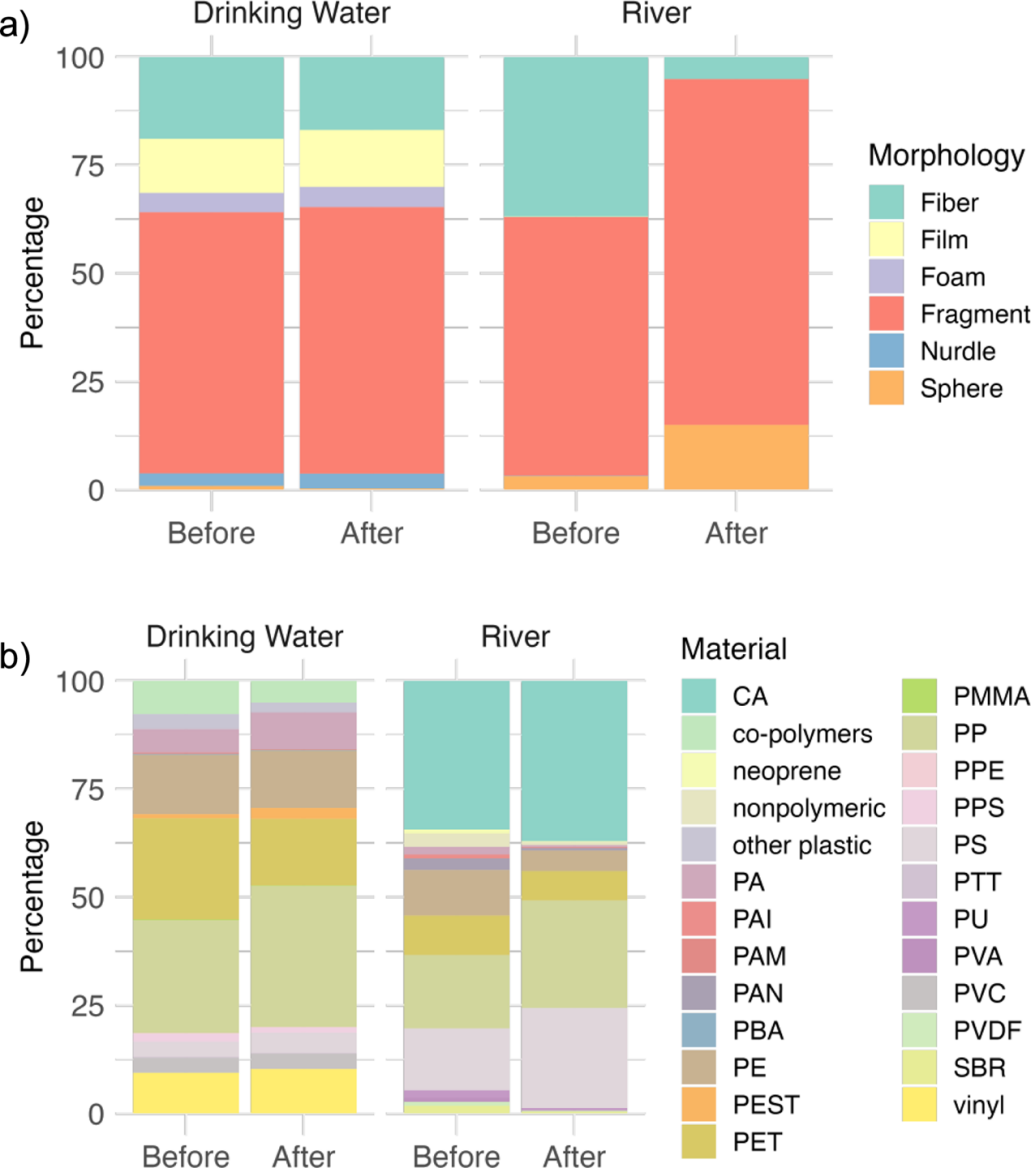
Changes in morphological (a) and polymeric (b) composition of microplastics by count in rivers and drinking water before and after size rescaling. Co-polymers, nonpolymeric, and other plastics have been lumped via the taxonomy for visualization. (CA: cellulose acetate, PA: polyamide, PAI: polyamide-imides, PAM: polyacrylamide, PAN: polyacrylonitrile, PBA: polybutylene terephthalate, PE: polyethylene , PEST: polyester, PET: polyethylene terephthalate, PMMA: poly(methyl methacrylate), PP: polypropylene, PPE: polyphenylene ether, PPS: polyphenylene sulfide, PS: polystyrene, PTT: polytrimethylene terephthalate, PU: polyurethane, PVA: polyvinyl acetate, PVC: polyvinyl chloride, PVDF: polyvinylidene fluoride, and SBR: styrene–butadiene rubber).

As mentioned previously, correcting concentrations by rescaling particle size ranges also has implications for shifts in categorical variable proportions when comparing multiple studies. Some of the notable changes in morphological proportions after being run through MaTCH are an apparent 11% decrease in fibers and an 18% increase in nurdles for drinking water studies, as illustrated below (Figure 4a). Even greater changes were found for riverine studies, with an apparent 86% decrease in the number of fibers and a 34% increase in the number of fragments. Lower changes in drinking water morphological proportions with size rescaling are likely due to the tendency for drinking water studies to focus on a similar size range to the full distribution (1–5000 μm), meaning that their correction factors will be smaller than those only analyzing larger microplastics. Corrected concentrations also shifted material proportions (Figure 4b). Notably, corrected drinking water concentrations saw an apparent decrease in PET (polyethylene terephthalate) by 34% and an increase in PEST (polyester) by 164%. For riverine studies, we saw an apparent 61% increase in PS (polystyrene) and a 55% decrease in PE (polyethylene).

Examining differences in morphological makeup before and after being run through MaTCH may help to provide insight into some methodological limitations. Though values obtained through MaTCH do contain a high degree of uncertainty, we believe that the analytical output is representative of the mean tendency. As drinking water studies tend to analyze smaller particles, it is possible that they are underreporting fibers at a higher proportion than environmental monitoring studies. Multiple reviews have found fibers to be the most abundant microplastic morphology in the environment,^53−55^ meaning our findings may highlight decreasing recovery of selective morphologies as the analyzed size range decreases, assuming a constant PSD across morphologies. Given that most studies define microplastic size by the longest axis, it follows that fibers and their very high length to width ratio have a lower projected surface area as compared to other morphologies. Therefore, fibers are more likely to be beneath the limit of diffraction for common spectral analysis methods such as Fourier transform infrared spectroscopy (FTIR) than their morphological counterparts within the same size class when classes are defined on the basis of the longest principal axes. We also see a much higher proportion of fibers reported in riverine data that target larger particle sizes than we see in drinking water studies or in size range-adjusted concentrations of riverine samples.

Conversely, there may also exist variable recovery rates between morphologies based on the sampling method, regardless of the studied size range. Net-based sampling and defining size classes by mesh size but defining particle size by the longest axis can propose a problem for morphologies such as fibers. Fibers are unique due to their flexibility and previously mentioned high length to width ratio, giving them the potential to deform and slip through nets, despite the length of the fiber being larger than the mesh size. This same issue of variable recovery rates applies to any sample that undergoes sieving, meaning this would lead to the opposite effect as mentioned in the prior paragraph and would, in fact lead us to believe riverine studies that utilize net-based sampling and undergo sieving would underreport fibers. Future work should investigate dominant sources of preferential recovery due to methodology based on morphology and fibers in particular.

Semantic proportions changing at different rates across size distributions in these examples indicate an interesting proposition: different PSDs may need to be developed for different morphologies or materials to traverse between semantic and quantitative data structures more accurately as well as to better characterize methodological limitations such as those mentioned above. Biases in modes of preferential environmental transport, limits of existing sampling and analytical methods, or reflections of differences in actual total abundances of various microplastic particle types across size distributions (possibly due to differing degradation pathways between materials or morphologies) may lead to a wide variety of PSDs when developed empirically rather than theoretically or with limited datasets. Additionally, some degree of particle level data reporting is recommended moving forward to promote transparency and enable direct comparisons between studies by allowing for more robust concentration rescaling. Though most groups do not record particle level data for an entire sample, typically, some subset of particles is taken to be characterized via spectroscopy, and even the sharing of morphological or size information on more robustly characterized subsets would strengthen existing empirical relationships. This type of reporting allows for the association of different particle characteristics (e.g., size distributions of individual morphologies can be analyzed). We believe this is an important topic to be investigated in future studies and that the use of MaTCH will help with identifying and pursuing these insights.

### 3.3 Methodological Limitations

The application of data-transforming operations posthoc is not without its limitations. While the application of different alpha values that reflect varying PSDs across environmental compartments is an improvement over assuming constant PSDs universally, these compartment-specific values are limited by the quality, amount, and specificity of the data with which empirical models were fit. More broadly, empirical models, such as those included in this study, are limited by the volume and quality of data used to develop them. Further work will be required to evaluate whether we observe these trends in systems not used in the development of the empirical models themselves and preferentially fit models to data from individual systems. Additionally, this model currently does not consider methodological variability, including from sampling apparatus or sample processing steps. Such variability should be explored to investigate if we observe significantly different trends as a function of study design that can then be used to further improve interstudy harmonization. One final limitation worth noting is the extrapolation of particle size ranges beyond what is studied. Though these empirically developed distributions are useful in improving harmonization across existing studies, it remains ideal for future studies to investigate the largest possible particle size range to avoid introducing unnecessary bias.

This study attempts to maximize transparency and capture the full range of uncertainties associated with such empirical models. However, we recognize the large range of uncertainty derived from data transformations such as those utilized here and encourage future studies to build off this framework to further reduce uncertainty. This tool is a step toward increased utilization of existing and new empirical harmonization methods to make improvements toward (1) creating more comparable results from misaligned monitoring studies and (2) informing future study designs from existing data on similar systems.

### 3.4 Implications and Next Steps

We believe our findings highlight a general need for more careful consideration of dataset characteristics when comparing between studies in the future. Moving forward, we propose building off existing non-semantic data harmonization frameworks with larger quantities of empirical data to better establish these fundamental properties of plastic characterization. This includes the relationship between morphology and *L:W:H*, as well as polymer–density relationships. The development of PSDs for nanoplastics could also be incorporated into the proposed framework to extend the reach of the potential particle size rescaling. Refining these distributions with respect to more detailed and higher volumes of data to describe more accurately what we observe in the environment is an important area of research moving forward. We hope this goal can be achieved by utilizing open-source, user-supported databases such as the one described in this study.

We believe these findings illustrate not just the need for data alignment in future studies and meta-analyses but also the interdependency of data harmonization techniques. Each step in the proposed algorithm is necessary to harmonize the quantity and characteristic makeup of microplastic samples. While a universal framework for microplastics data reporting does not currently exist, we will continue to develop and refine data harmonization algorithms to leverage valuable existing data with proper consideration of possible biases.

## Data Availability

Data and code used to generate figures, algorithm, and the Shiny App are openly available on Zenodo at: https://zenodo.org/records/13901058, GitHub at: https://github.com/hannahhapich/MaTCH/releases/tag/Publication.

## Supporting Information Available

Additional equations, database diagram, additional case study, and meta-analysis study information (PDF)
User guide for accompanying R Shiny web tool (PDF)
