# Supplementary material for: Microplastics and Trash Cleaning and Harmonization (MaTCH): Semantic Data Ingestion and Harmonization Using Artificial Intelligence
Source: Environ Sci Technol. 2024 Nov 11;58(46):20502–12. doi: 10.1021/acs.est.4c02406 (PMC11580164; doi:10.1021/acs.est.4c02406)
Supplement: Supplementary file 1 — es4c02406_si_001.pdf [file es4c02406_si_001.pdf]

Supporting Information

Microplastics and Trash Cleaning and Harmonization (MaTCH): Semantic Data Ingestion and Harmonization Using Artificial Intelligence (AI)

Authorship List and Affiliations

Hannah Hapich[1]*, Win Cowger[1,2], Andrew B Gray[1]

[1]University of California, Riverside, Riverside, California 92521

[2]Moore Institute for Plastic Pollution Research, Long Beach, California 90803

*Corresponding author: hannahhapich@gmail.com

Address: 900 University Avenue, Riverside, CA 92521

Description

Additional equations, database diagram, additional case study, and meta-analysis study information

Summary

13 pages, 3 figures, 2 equations, and 1 table

Contents

Figure S1: DB diagram of relational database structure……………………………………………………………S2

Relational Table Database: Use Cases………………………………………………………………………………S3

Equation S1: Inverse Power Law Function……………………………………………………………S3
Equation S2: Correction Factor…………………………………………………………………..…S3
Figure S2: Average comparability for morphology and material……………………………..…S4
Model Testing: Micro vs. Macro Roadway Debris Meta-Analysis Harmonization………………S5
Methods…………………………………………………………………………………………S5
Results……………………………………………………………………..…………………S6
Figure S3: Stacked bar plots of morphology and material……………………………………….…S8
Table S1: Studies included in meta-analysis of microplastic occurrence in drinking water and freshwater ecosystems ………………………………………………………………………………………S8

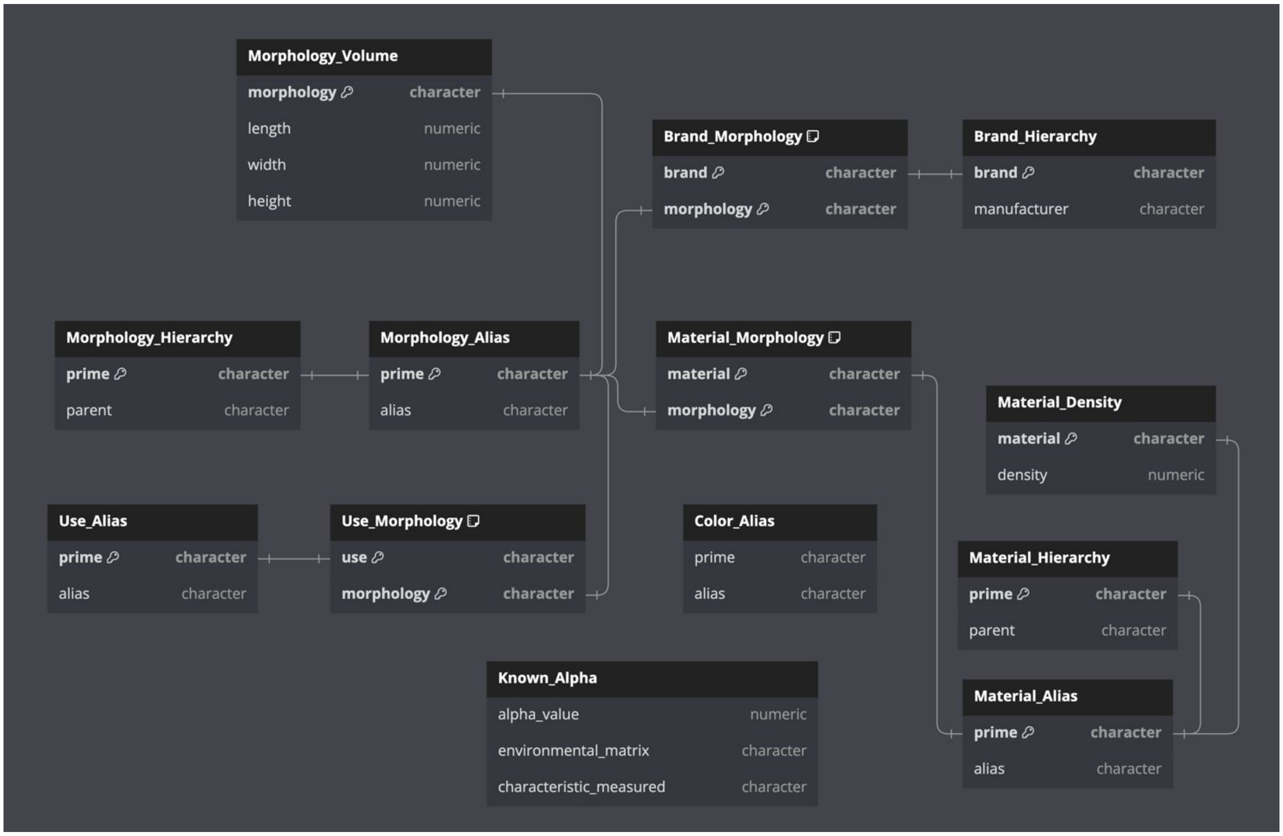

Figure S1: *DB diagram of relational database structure, including microplastics and use cases. The key icons indicate key terms used to link relational tables. An interactive version of this diagram is available at: https://dbdiagram.io/d/MaTCH-64e7b4be02bd1c4a5e5d44fc*

Relational Table Database: Use Cases

In addition to microplastic data integration, we have also integrated a new category of "use cases" to the suite of relational tables (Fig. S1). We define a use case as any term that does not describe a particular morphology or material type, but rather describes the usage related to that particular object. Such terms were previously excluded from our database as "unclassifiable" terms as they do not provide any qualitative data to inform mitigation of mismanaged waste (e.g., "fishing related", "construction materials", etc.). However, with the common usage of such terms and their ability to help inform source, we added two relational tables to our database to describe use cases. A prime use case data table relates synonymous terms (e.g., fishing-gear and fishing-related), and a morphology-use relational table that links each morphology to its associated use (e.g., fishing line to fishing-gear). These tables are termed "Use_Alias" and "Use_Morphology" in the DB diagram in Fig. S1, illustrating their place in this relational database framework.

$$y = bx^{-\alpha}$$

Equation S1: *Inverse Power Law Function* [1]

$$CF = \frac{{L_{UL,D}}^{1-\alpha} - {L_{LL,D}}^{1-\alpha}}{{L_{UL,M}}^{1-\alpha} - {L_{LL,M}}^{1-\alpha}}$$

Equation S2: *Correction Factor (CF = correction factor, α from equation S1, $L_{UL,D}$= upper limit default length, $L_{LL,D}$= lower limit default length, $L_{UL,M}$= upper limit measured length, $L_{LL,M}$= upper*

limit measured length) [2] *Note: When α = 1, Equation S2 results in a division by zero error. In this case, rather than using α = 1, MaTCH will take the limit of α to solve for CF.*

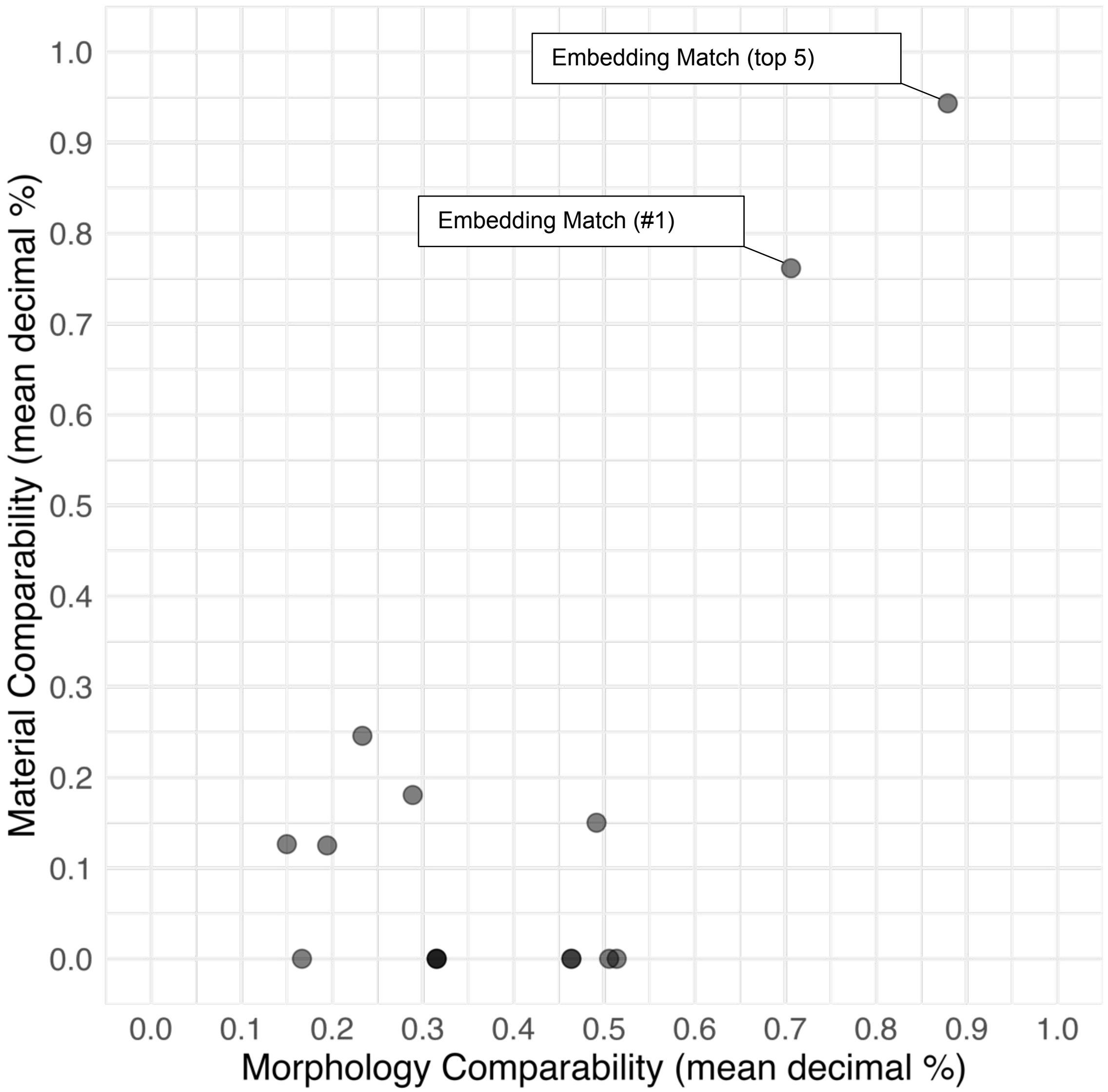

Figure S2: *Average comparability for each microplastic study by morphology and material categories. Morphology comparability is plotted on the x-axis, and material comparability is plotted on the y axis.*

Model Testing: Micro vs. Macro Roadway Debris Meta Analysis Harmonization

Methods

To illustrate data variability when reported as particle count vs. mass, we analyzed data from studies on both micro and macro debris in urban roadways. Macro debris data was obtained from a litter accumulation study on urban roadways in Southern California that reported individual particle information (n = 2341 pieces of litter) [3]. Microparticle—including both plastic and non-plastic micro debris—data was obtained from Järlskog et al. 2022, a study of traffic-derived non-exhaust particulates on a roadway in Sweden, utilizing SEM/EDX to analyze particle material [4]. Both studies were chosen due to their similarity in ranges of materials studied (both plastic and non-plastic), similar environmental media (curbside roadways), and data availability.

Microparticle data was reported for two size bins (2-20 μm and 20-125 μm). Proportions of material and morphology for each size bin were combined in R with a PDF (α = 1.6) for both the 2-20 μm and 20-125 μm size bins to generate particle data for reported average sample concentrations, as outlined in section 2.2.1 in the main text. For the purposes of this analysis, mineral particles have been excluded. No morphological data was provided, though materials reported suggested the origin of microparticles and assumptions could be made on their corresponding morphology types. Glass, metallic, organic, asphalt, and tire wear particles were assumed to be fragments, and paint were assumed films.

Semantic data from both studies were merged with our database and embeddings were generated when needed. Accuracy of embedding matches were also assessed. Any terms

matched incorrectly were adjusted to better reflect the reported data. Micro particle masses were calculated according to methods outlined in section 2.2.1. Mass of macro debris was calculated using mass estimates from a meta-analysis of measured particles, also discussed further in section 2.2.1 [3]. Concentrations were normalized to count/m^2 for both studies. Average particle mass for each study was used to convert count/m^2 to g/m^2.

Results

Our review of roadway studies for macro and micro particles created a more comparable metric of abundance by converting particle count to mass. The study on macro debris found 0.06 particles/m^2 on curbside roadways. Micro particles were found to have an average of 2.34 $x10^{11}$ particles/m^2 on curbsides. Using MaTCH to convert to mass, we see 1.56 g/m^2 curbside macro debris and 615 g/m^2 curbside micro particle. Combined, this gives us 617 g/m^2 curbside debris of all size ranges, with a vast majority coming from micro particles.

Changes in morphology and material proportions when reported by count vs by mass for all particles are shown below in Figures S3a and S3b. Just as size rescaled concentrations shift semantic proportions from section 3.2 in the main text, concentrations rescaled on the basis of mass rather than count also resulted in discernable changes. For macro debris morphologies when traversing from count to mass, "other" increased from 18.36% to 52.86%, and "wrappers" decreased from 18.07% to 9.21% (Fig. S3a). Less dramatic changes were seen with respect to macro debris materials, with "plastic" decreasing from 56.64% by count to 49.52% by mass and "glass" increasing from 1.54% by count to 9.00% by mass (Fig. S3a). Micro debris particle characteristics shifted as well. For morphologies, "fragment" increased from 95.82% to 99.88%, while "film" decreased from 4.18% to 0.12% (Fig. S3b) when represented as

count and mass. respectively. Strong shifts were seen in materials as the larger particles were characterized as “tire wear” or “asphalt”, while smaller particles could not be discerned and both categories were combined into “tire wear & asphalt.” We can see this clearly in Fig. S3b with “tire wear & asphalt” decreasing dramatically from 82.96% by count to only 10.73% by mass. Conversely, “tire wear” increased from 1.12% by count to 40.35% by mass, and “asphalt” increased from 0.71% to 40.96%.

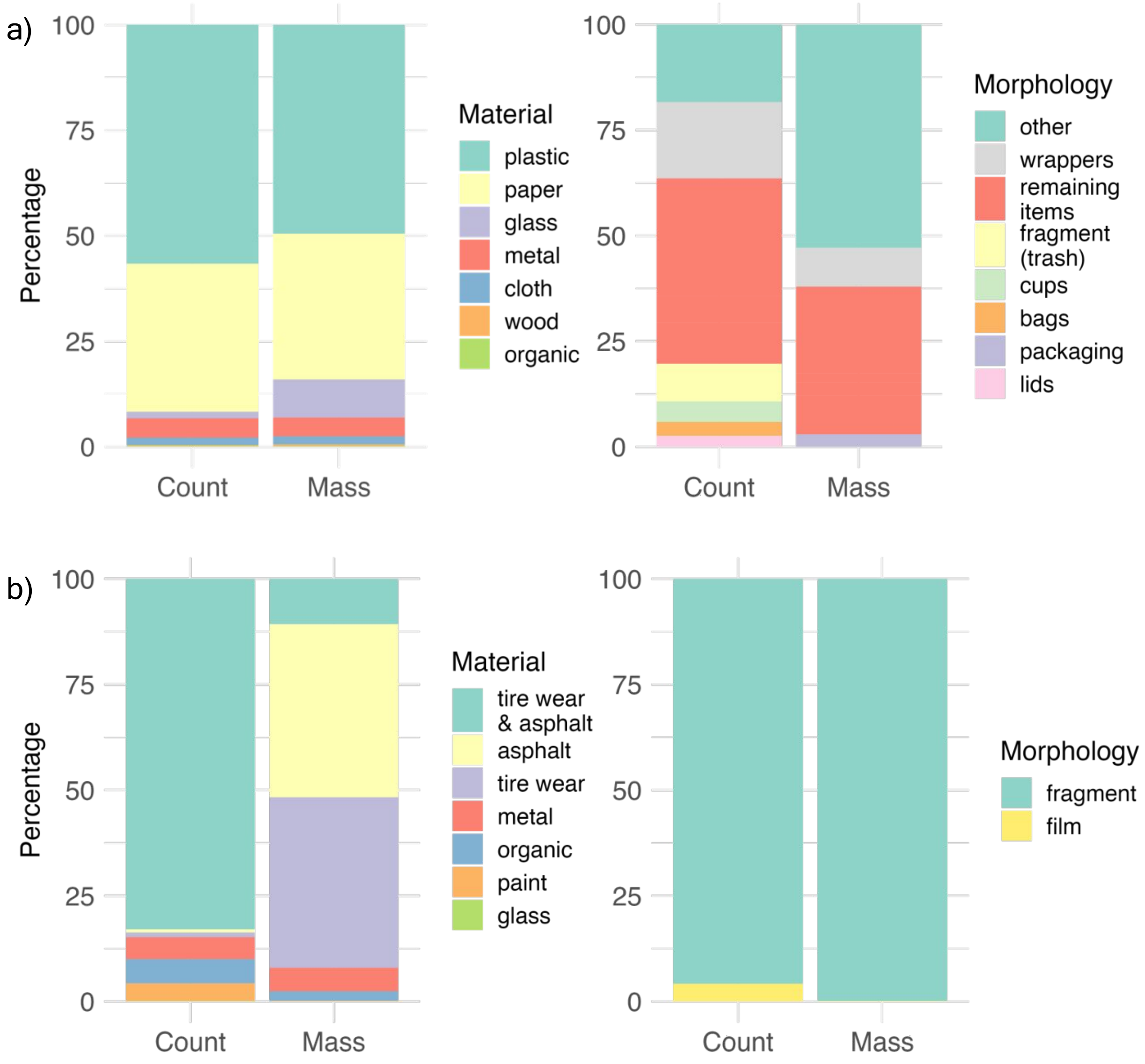

Figure S3: *Changes in material (left) and morphological (right) proportions of macro debris (S3a) and micro debris (S3b) when represented by count (left) and by mass (right)*

Table S1: Studies included in meta-analysis of microplastic occurrence in drinking water and freshwater ecosystems

DOI	Study Matrix	Number of Datapoints	Reference
10.1016/j.sciotenv.2018.08.102	Drinking Water	6	[5]
10.1371/journal.pone.0236838	Drinking Water	8	[6]
10.1016/j.impact.2021.100302	Drinking Water	9	[7]
10.1016/j.scitotenv.2020.140236	Drinking Water	4	[8]
10.1016/j.chemosphere.2020.126493	Drinking Water	38	[9]
10.1007/s11356-021-13769-x	Drinking Water	8	[10]
10.1016/j.scitotenv.2022.154015	Drinking Water	1	[11]
10.1016/j.envpol.2020.114227	Drinking Water	42	[12]
10.1016/j.scitotenv.2019.134520	Drinking Water	1	[13]
10.1016/j.jwpe.2020.101884	Drinking Water	23	[14]
10.1039/c6ay01184e	Drinking Water	2	[15]
10.18520/cs/v117/i11/1874-1879	Drinking Water	6	[16]
10.1016/j.scitotenv.2021.150545	Drinking Water	63	[17]
10.1016/j.etap.2018.12.009	Drinking Water	7	[18]
10.1016/j.jhazmat.2021.125347	Drinking Water	5	[19]
10.1016/j.chemosphere.2020.126612	Drinking Water	15	[20]
10.1007/s11356-021-13220-1	Drinking Water	5	[21]
10.1016/j.sciotenv.2020.143421	Drinking Water	1	[22]
10.3389/fchem.2018.00407	Drinking Water	27	[23]
10.22630/PNIKS.2019.28.4.59	Drinking Water	12	[24]
10.1371/journal.pone.0194970	Drinking Water	27	[25]
10.1016/j.watres.2017.11.011	Drinking Water	4	[26]
10.1016/j.sciotenv.2018.08.178	Drinking Water	24	[27]
10.1016/j.envres.2022.112855	Drinking Water	1	[28]
10.1007/s11356-021-12467-y	Drinking Water	1	[29]
10.3390/w12113115	Drinking Water	1	[30]
10.1016/j.chemosphere.2021.132587	Drinking Water	4	[31]
10.1016/j.envpol.2021.117524	Drinking Water	6	[32]
http://dce2.au.dk/pub/SR291.pdf	Drinking Water	18	[33]
10.21203/rs.3.rs-242504/v1	Drinking Water	3	[34]
10.11001/jksww.2020.34.5.357	Drinking Water	2	[35]

10.1016/j.scitotenv.2019.02.431	Drinking Water	21	[36]
10.1016/j.cej.2020.128381	Drinking Water	23	[37]
10.1007/s11783-021-1492-5	Drinking Water	4	[38]
10.1021/acs.est.9b01517	Drinking Water	2	[39]
10.1016/j.cotox.2021.09.003	Drinking Water	34	[40]
10.1016/j.cofs.2021.02.011	Drinking Water	8	[41]
10.3390/app112110109	Drinking Water	112	[42]
10.1016/j.scitotenv.2021.148001	Drinking Water	14	[43]
10.3390/environments8120138	Drinking Water	3	[44]
doi.org/10.1007/s11356-021-12898-7	Effluent	6	[45]
doi.org/10.1016/j.scitotenv.2019.02.028	Freshwater Sediment	36	[46]
doi.org/10.1016/j.jwpe.2020.101640	Freshwater Sediment	4	[47]
doi.org/10.1016/j.marpolbul.2017.07.028	Freshwater Surface	1	[48]
doi.org/10.5894/rgci194	Freshwater Surface	18	[49]
doi.org/10.1002/eap.2429	Freshwater Surface	27	[50]
doi.org/10.1021/acs.est.6b02917	Freshwater Surface	104	[51]
doi.org/10.1021/es5036317	Freshwater Surface	16	[52]
doi.org/10.1021/acs.est.9b03850	Freshwater Surface	3	[53]
doi.org/10.1021/acsestwater.1c00072	Freshwater Surface	54	[54]
doi.org/10.1021/acs.est.1c03019	Freshwater Surface	16	[55]
doi.org/10.1021/es503610r	Freshwater Surface	2	[56]
doi.org/10.1016/j.envpol.2018.06.033	Freshwater Surface	54	[57]
doi.org/10.1021/acs.est.9b04896	Freshwater Surface	34	[58]
doi.org/10.5203/pmuser.201730578	Freshwater Surface	12	[59]
doi.org/10.1002/ecs2.1556	Freshwater Surface	20	[60]
doi.org/10.1016/j.watres.2018.10.013	Freshwater Surface	160	[61]

(1) Kooi, M.; Koelmans, A. A. Simplifying Microplastic via Continuous Probability Distributions for Size, Shape,and Density. *Environ. Sci. Technol. Lett.* **2019**, *6* (9), 551–557. https://doi.org/10.1021/acs.estlett.9b00379.

(2) Koelmans, A. A.; Redondo-Hasselerharm, P. E.; Mohamed Nor, N. H.; Kooi, M. Solving the Nonalignment of Methods and Approaches Used in Microplastic Research to Consistently Characterize Risk. *Environ. Sci. Technol.* **2020**, *54* (19), 12307–12315. https://doi.org/10.1021/acs.est.0c02982.

(3) Cowger, W.; Gray, A.; Hapich, H.; Osei-Enin, J.; Olguin, S.; Huynh, B.; Nogi, H.; Singh, S.; Brownlee, S.; Fong, J.; Lok, T.; Singer, G.; Ajami, H. Litter Origins, Accumulation Rates, and Hierarchical Composition on Urban Roadsides of the Inland Empire, California. *Environ. Res. Lett.* **2022**, *17* (1). https://doi.org/10.1088/1748-9326/ac3c6a.

(4) Järlskog, I.; Jaramillo-Vogel, D.; Rausch, J.; Gustafsson, M.; Strömvall, A. M.; Andersson-Sköld, Y. Concentrations of Tire Wear Microplastics and Other Traffic-Derived Non-Exhaust Particles in the Road Environment. *Environ. Int.* **2022**, *170* (June). https://doi.org/10.1016/j.envint.2022.107618.

(5) Pivokonsky, M.; Cermakova, L.; Novotna, K.; Peer, P.; Cajthaml, T.; Janda, V.

Occurrence of Microplastics in Raw and Treated Drinking Water. *Sci. Total Environ.* **2018**, *643*, 1644–1651. https://doi.org/10.1016/j.scitotenv.2018.08.102.

(6) Danopoulos, E.; Twiddy, M.; Rotchell, J. M. Microplastic Contamination of Drinking Water: A Systematic Review. *PLoS One* **2020**, *15* (7 July), 1–23. https://doi.org/10.1371/journal.pone.0236838.

(7) Mortensen, N. P.; Fennell, T. R.; Johnson, L. M. Unintended Human Ingestion of Nanoplastics and Small Microplastics through Drinking Water, Beverages, and Food Sources. *NanoImpact* **2021**, *21* (January), 100302. https://doi.org/10.1016/j.impact.2021.100302.

(8) Pivokonský, M.; Pivokonská, L.; Novotná, K.; Čermáková, L.; Klimtová, M. Occurrence and Fate of Microplastics at Two Different Drinking Water Treatment Plants within a River Catchment. *Sci. Total Environ.* **2020**, *741*. https://doi.org/10.1016/j.scitotenv.2020.140236.

(9) Tong, H.; Jiang, Q.; Hu, X.; Zhong, X. Occurrence and Identification of Microplastics in Tap Water from China. *Chemosphere* **2020**, *252*, 126493. https://doi.org/10.1016/j.chemosphere.2020.126493.

(10) Shen, M.; Zeng, Z.; Wen, X.; Ren, X.; Zeng, G.; Zhang, Y.; Xiao, R. Presence of Microplastics in Drinking Water from Freshwater Sources: The Investigation in Changsha, China. *Environ. Sci. Pollut. Res.* **2021**, *28* (31), 42313–42324. https://doi.org/10.1007/s11356-021-13769-x.

(11) Jung, J. W.; Kim, S.; Kim, Y. S.; Jeong, S.; Lee, J. Tracing Microplastics from Raw Water to Drinking Water Treatment Plants in Busan, South Korea. *Sci. Total Environ.* **2022**, *825*, 154015. https://doi.org/10.1016/j.scitotenv.2022.154015.

(12) Shruti, V. C.; Pérez-Guevara, F.; Kutralam-Muniasamy, G. Metro Station Free Drinking Water Fountain- A Potential "Microplastics Hotspot" for Human Consumption. *Environ. Pollut.* **2020**, *261*. https://doi.org/10.1016/j.envpol.2020.114227.

(13) Wang, Z.; Lin, T.; Chen, W. Occurrence and Removal of Microplastics in an Advanced Drinking Water Treatment Plant (ADWTP). *Sci. Total Environ.* **2020**, *700*, 134520. https://doi.org/10.1016/j.scitotenv.2019.134520.

(14) Zhou, X. jun; Wang, J.; Li, H. yan; Zhang, H. min; Hua-Jiang; Zhang, D. L. Microplastic Pollution of Bottled Water in China. *J. Water Process Eng.* **2021**, *40* (November 2020), 101884. https://doi.org/10.1016/j.jwpe.2020.101884.

(15) Wiesheu, A. C.; Anger, P. M.; Baumann, T.; Niessner, R.; Ivleva, N. P. Raman Microspectroscopic Analysis of Fibers in Beverages. *Anal. Methods* **2016**, *8* (28), 5722–5725. https://doi.org/10.1039/c6ay01184e.

(16) Singhal, G.; Bansod, B.; Mathew, L.; Goswami, J.; Choudhury, B. U.; Raju, P. L. N. Comparison of Parametric and Non-Parametric Methods for Chlorophyll Estimation Based on High-Resolution UAV Imagery. *Curr. Sci.* **2019**, *117* (11), 1874–1879. https://doi.org/10.18520/cs/v117/i11/1874-1879.

(17) Wu, J.; Zhang, Y.; Tang, Y. Fragmentation of Microplastics in the Drinking Water Treatment Process - A Case Study in Yangtze River Region, China. *Sci. Total Environ.* **2022**, *806*, 150545. https://doi.org/10.1016/j.scitotenv.2021.150545.

(18) Di, M.; Liu, X.; Wang, W.; Wang, J. Manuscript Prepared for Submission to Environmental Toxicology and Pharmacology Pollution in Drinking Water Source Areas: Microplastics in the Danjiangkou Reservoir, China. *Environ. Toxicol. Pharmacol.* **2019**, *65* (August 2018), 82–89. https://doi.org/10.1016/j.etap.2018.12.009.

(19) Sarkar, D. J.; Das Sarkar, S.; Das, B. K.; Praharaj, J. K.; Mahajan, D. K.; Purokait, B.; Mohanty, T. R.; Mohanty, D.; Gogoi, P.; Kumar V, S.; Behera, B. K.; Manna, R. K.; Samanta, S. Microplastics Removal Efficiency of Drinking Water Treatment Plant with Pulse Clarifier. *J. Hazard. Mater.* **2021**, *413* (November 2020), 125347. https://doi.org/10.1016/j.jhazmat.2021.125347.

(20) Shen, M.; Song, B.; Zhu, Y.; Zeng, G.; Zhang, Y.; Yang, Y.; Wen, X.; Chen, M.; Yi, H. Removal of Microplastics via Drinking Water Treatment: Current Knowledge and Future Directions. *Chemosphere* **2020**, *251*, 126612. https://doi.org/10.1016/j.chemosphere.2020.126612.

(21) Dalmau-Soler, J.; Ballesteros-Cano, R.; Boleda, M. R.; Paraira, M.; Ferrer, N.; Lacorte, S. Microplastics from Headwaters to Tap Water: Occurrence and Removal in a Drinking Water Treatment Plant in Barcelona Metropolitan Area (Catalonia, NE Spain). *Environ. Sci. Pollut. Res.* **2021**, *28* (42), 59462–59472. https://doi.org/10.1007/s11356-021-13220-1.

(22) Weber, F.; Kerpen, J.; Wolff, S.; Langer, R.; Eschweiler, V. Investigation of Microplastics Contamination in Drinking Water of a German City. *Sci. Total Environ.* **2021**, *755*, 143421. https://doi.org/10.1016/j.scitotenv.2020.143421.

(23) Mason, S. A.; Welch, V.; Neratko, J. Synthetic Polymer Contamination in Global Drinking Water. *Fredonia* **2017**, 1–17.

(24) Paredes, M.; Castillo, T.; Viteri, R.; Fuentes, G.; Bodero, E. Microplastics in the Drinking Water of the Riobamba City, Ecuador. *Sci. Rev. Eng. Environ. Sci.* **2019**, *28* (4), 653–663. https://doi.org/10.22630/PNIKS.2019.28.4.59.

(25) Kosuth, M.; Mason, S. A.; Wattenberg, E. V. Anthropogenic Contamination of Tap Water, Beer, and Sea Salt. *PLoS One* **2018**, *13* (4), 1–18. https://doi.org/10.1371/journal.pone.0194970.

(26) Schymanski, D.; Goldbeck, C.; Humpf, H. U.; Fürst, P. Analysis of Microplastics in Water by Micro-Raman Spectroscopy: Release of Plastic Particles from Different Packaging into Mineral Water. *Water Res.* **2018**, *129*, 154–162. https://doi.org/10.1016/j.watres.2017.11.011.

(27) Mintenig, S. M.; Löder, M. G. J.; Primpke, S.; Gerdts, G. Low Numbers of Microplastics Detected in Drinking Water from Ground Water Sources. *Sci. Total Environ.* **2019**, *648*, 631–635. https://doi.org/10.1016/j.scitotenv.2018.08.178.

(28) Shi, J.; Dong, Y.; Shi, Y.; Yin, T.; He, W.; An, T.; Tang, Y.; Hou, X.; Chong, S.; Chen, D.; Qin, K.; Lin, H. Groundwater Antibiotics and Microplastics in a Drinking-Water Source Area, Northern China: Occurrence, Spatial Distribution, Risk Assessment, and Correlation. *Environ. Res.* **2022**, *210* (August 2021), 112855. https://doi.org/10.1016/j.envres.2022.112855.

(29) Pittroff, M.; Müller, Y. K.; Witzig, C. S.; Scheurer, M.; Storck, F. R.; Zumbülte, N. Microplastic Analysis in Drinking Water Based on Fractionated Filtration Sampling and Raman Microspectroscopy. *Environ. Sci. Pollut. Res.* **2021**, *28* (42), 59439–59451. https://doi.org/10.1007/s11356-021-12467-y.

(30) Ferraz, M.; Bauer, A. L.; Valiati, V. H.; Schulz, U. H. Microplastic Concentrations in Raw and Drinking Water in the Sinos River, Southern Brazil. *Water (Switzerland)* **2020**, *12* (11), 1–10. https://doi.org/10.3390/w12113115.

(31) Cherniak, S. L.; Almuhtaram, H.; McKie, M. J.; Hermabessiere, L.; Yuan, C.; Rochman, C. M.; Andrews, R. C. Conventional and Biological Treatment for the Removal of Microplastics from Drinking Water. *Chemosphere* **2022**, *288* (P2), 132587. https://doi.org/10.1016/j.chemosphere.2021.132587.

(32) Siegel, H.; Fischer, F.; Lenz, R.; Fischer, D.; Jekel, M.; Labrenz, M. Identification and Quantification of Microplastic Particles in Drinking Water Treatment Sludge as an Integrative Approach to Determine Microplastic Abundance in a Freshwater River. *Environ. Pollut.* **2021**, *286* (June), 117524. https://doi.org/10.1016/j.envpol.2021.117524.

(33) Strand, J.; Feld, L.; Murphy, F.; Mackevica, A.; Hartmann, N. B. *Analysis of Microplastic Particles*; 2018.

(34) Adib, D.; Mafigholami, R.; Tabeshkia, H. Identification of Microplastics in Conventional Drinking Water Treatment Plants in Tehran, Iran. *J. Environ. Heal. Sci. Eng.* **2021**, *19* (2),

1817–1826. https://doi.org/10.1007/s40201-021-00737-3.
(35) Choi, B. A Mini-Review on Microplastics in Drinking Water Treatment Processes. **2020**, *34* (5), 357–371.
(36) Novotna, K.; Cermakova, L.; Pivokonska, L.; Cajthaml, T.; Pivokonsky, M. Microplastics in Drinking Water Treatment – Current Knowledge and Research Needs. *Sci. Total Environ.* **2019**, *667*, 730–740. https://doi.org/10.1016/j.scitotenv.2019.02.431.
(37) Cheng, Y. L.; Kim, J. G.; Kim, H. Bin; Choi, J. H.; Fai Tsang, Y.; Baek, K. Occurrence and Removal of Microplastics in Wastewater Treatment Plants and Drinking Water Purification Facilities: A Review. *Chem. Eng. J.* **2021**, *410* (January), 128381. https://doi.org/10.1016/j.cej.2020.128381.
(38) Xue, J.; Samaei, S. H. A.; Chen, J.; Doucet, A.; Ng, K. T. W. What Have We Known so Far about Microplastics in Drinking Water Treatment? A Timely Review. *Front. Environ. Sci. Eng.* **2022**, *16* (5). https://doi.org/10.1007/s11783-021-1492-5.
(39) Cox, K. D.; Covernton, G. A.; Davies, H. L.; Dower, J. F.; Juanes, F.; Dudas, S. E. Human Consumption of Microplastics. *Environ. Sci. Technol.* **2019**, *53* (12), 7068–7074. https://doi.org/10.1021/acs.est.9b01517.
(40) Kirstein, I. V.; Gomiero, A.; Vollertsen, J. Microplastic Pollution in Drinking Water. *Curr. Opin. Toxicol.* **2021**, *28*, 70–75. https://doi.org/10.1016/j.cotox.2021.09.003.
(41) Oßmann, B. E. Microplastics in Drinking Water? Present State of Knowledge and Open Questions. *Curr. Opin. Food Sci.* **2021**, *41*, 44–51. https://doi.org/10.1016/j.cofs.2021.02.011.
(42) Sol, D.; Laca, A.; Laca, A.; Díaz, M. Microplastics in Wastewater and Drinking Water Treatment Plants: Occurrence and Removal of Microfibres. *Appl. Sci.* **2021**, *11* (21). https://doi.org/10.3390/app112110109.
(43) Huang, S.; Peng, C.; Wang, Z.; Xiong, X.; Bi, Y.; Liu, Y.; Li, D. Spatiotemporal Distribution of Microplastics in Surface Water, Biofilms, and Sediments in the World's Largest Drinking Water Diversion Project. *Sci. Total Environ.* **2021**, *789*, 148001. https://doi.org/10.1016/j.scitotenv.2021.148001.
(44) Luqman, A.; Nugrahapraja, H.; Wahyuono, R. A.; Islami, I.; Haekal, M. H.; Fardiansyah, Y.; Putri, B. Q.; Amalludin, F. I.; Rofiqa, E. A.; Götz, F.; Wibowo, A. T. Microplastic Contamination in Human Stools, Foods, and Drinking Water Associated with Indonesian Coastal Population. *Environ. - MDPI* **2021**, *8* (12), 1–9. https://doi.org/10.3390/environments8120138.
(45) Prajapati, S.; Beal, M.; Maley, J.; Brinkmann, M. Qualitative and Quantitative Analysis of Microplastics and Microfiber Contamination in Effluents of the City of Saskatoon Wastewater Treatment Plant. *Environ. Sci. Pollut. Res.* **2021**, *28* (25), 32545–32553. https://doi.org/10.1007/s11356-021-12898-7.
(46) Watkins, L.; McGrattan, S.; Sullivan, P. J.; Walter, M. T. The Effect of Dams on River Transport of Microplastic Pollution. *Sci. Total Environ.* **2019**, *664*, 834–840. https://doi.org/10.1016/j.scitotenv.2019.02.028.
(47) Christensen, N. D.; Wisinger, C. E.; Maynard, L. A.; Chauhan, N.; Schubert, J. T.; Czuba, J. A.; Barone, J. R. Transport and Characterization of Microplastics in Inland Waterways. *J. Water Process Eng.* **2020**, *38* (June), 101640. https://doi.org/10.1016/j.jwpe.2020.101640.
(48) Miller, R. Z.; Watts, A. J. R.; Winslow, B. O.; Galloway, T. S.; Barrows, A. P. W. Mountains to the Sea: River Study of Plastic and Non-Plastic Microfiber Pollution in the Northeast USA. *Mar. Pollut. Bull.* **2017**, *124* (1), 245–251. https://doi.org/10.1016/j.marpolbul.2017.07.028.
(49) Moore, C. J.; Lattin, G. L.; Zellers, A. F. Quantity and Type of Plastic Debris Flowing from Two Urban Rivers to Coastal Waters and Beaches of Southern California. *Rev. Gestão Costeira Integr.* **2011**, *11* (1), 65–73. https://doi.org/10.5894/rgci194.

(50) Vincent, A. E. S.; Hoellein, T. J. Distribution and Transport of Microplastic and Fine Particulate Organic Matter in Urban Streams. *Ecol. Appl.* **2021**, *31* (8), 1–16. https://doi.org/10.1002/eap.2429.
(51) Baldwin, A. K.; Corsi, S. R.; Mason, S. A. Plastic Debris in 29 Great Lakes Tributaries: Relations to Watershed Attributes and Hydrology. *Environ. Sci. Technol.* **2016**, *50* (19), 10377–10385. https://doi.org/10.1021/acs.est.6b02917.
(52) Yonkos, L. T.; Friedel, E. A.; Perez-Reyes, A. C.; Ghosal, S.; Arthur, C. D. Microplastics in Four Estuarine Rivers in the Chesapeake Bay, U.S.A. *Environ. Sci. Technol.* **2014**, *48* (24), 14195–14202. https://doi.org/10.1021/es5036317.
(53) Lenaker, P. L.; Baldwin, A. K.; Corsi, S. R.; Mason, S. A.; Reneau, P. C.; Scott, J. W. Vertical Distribution of Microplastics in the Water Column and Surficial Sediment from the Milwaukee River Basin to Lake Michigan. *Environ. Sci. Technol.* **2019**, *53* (21), 12227–12237. https://doi.org/10.1021/acs.est.9b03850.
(54) Haberstroh, C. J.; Arias, M. E.; Yin, Z.; Wang, M. C. Effects of Urban Hydrology on Plastic Transport in a Subtropical River. *ACS ES T Water* **2021**, *1* (8), 1714–1727. https://doi.org/10.1021/acsestwater.1c00072.
(55) Watkins, L.; Sullivan, P. J.; Walter, M. T. What You Net Depends on If You Grab: A Meta-Analysis of Sampling Method's Impact on Measured Aquatic Microplastic Concentration. *Environ. Sci. Technol.* **2021**, *55* (19), 12930–12942. https://doi.org/10.1021/acs.est.1c03019.
(56) McCormick, A.; Hoellein, T. J.; Mason, S. A.; Schluep, J.; Kelly, J. J. Microplastic Is an Abundant and Distinct Microbial Habitat in an Urban River. *Environ. Sci. Technol.* **2014**, *48* (20), 11863–11871. https://doi.org/10.1021/es503610r.
(57) Kapp, K. J.; Yeatman, E. Microplastic Hotspots in the Snake and Lower Columbia Rivers: A Journey from the Greater Yellowstone Ecosystem to the Pacific Ocean. *Environ. Pollut.* **2018**, *241*, 1082–1090. https://doi.org/10.1016/j.envpol.2018.06.033.
(58) Corcoran, P. L.; Belontz, S. L.; Ryan, K.; Walzak, M. J. Factors Controlling the Distribution of Microplastic Particles in Benthic Sediment of the Thames River, Canada. *Environ. Sci. Technol.* **2020**, *54* (2), 818–825. https://doi.org/10.1021/acs.est.9b04896.
(59) Warrack, S.; Challis, J. K.; Hanson, M. L.; Rennie, M. D. Microplastics Flowing into Lake Winnipeg: Densities, Sources, Flux, and Fish Exposures. *Proc. Manitoba's Undergrad. Sci. Eng. Res.* **2017**, *3*, 5–15. https://doi.org/10.5203/pmuser.201730578.
(60) McCormick, A. R.; Hoellein, T. J.; London, M. G.; Hittie, J.; Scott, J. W.; Kelly, J. J. Microplastic in Surface Waters of Urban Rivers: Concentration, Sources, and Associated Bacterial Assemblages. *Ecosphere* **2016**, *7* (11). https://doi.org/10.1002/ecs2.1556.
(61) Barrows, A. P. W.; Christiansen, K. S.; Bode, E. T.; Hoellein, T. J. A Watershed-Scale, Citizen Science Approach to Quantifying Microplastic Concentration in a Mixed Land-Use River. *Water Res.* **2018**, *147*, 382–392. https://doi.org/10.1016/j.watres.2018.10.013.
