# Supplementary material for: Microplastics and Trash Cleaning and Harmonization (MaTCH): Semantic Data Ingestion and Harmonization Using Artificial Intelligence
Source: Environ Sci Technol. 2024 Nov 11;58(46):20502–12. doi: 10.1021/acs.est.4c02406 (PMC11580164; doi:10.1021/acs.est.4c02406)
Supplement: Supplementary file 2 — es4c02406_si_002.pdf [file es4c02406_si_002.pdf]

Supporting Information

Microplastics and Trash Cleaning and Harmonization (MaTCH): Semantic Data Ingestion and Harmonization Using Artificial Intelligence (AI)

Authorship List and Affiliations

Hannah Hapich[1]*, Win Cowger[1,2], Andrew B Gray[1]

[1]University of California, Riverside, Riverside, California 92521

[2]Moore Institute for Plastic Pollution Research, Long Beach, California 90803

*Corresponding author: hannahhapich@gmail.com

Address: 900 University Avenue, Riverside, CA 92521

Description

User guide for accompanying R Shiny web tool

Summary

9 pages and 11 figures

Contents

1. **Getting Started……………………………………………………………………………………………….S2**
2. **Navigation……………………………………………………………………………………………………….S2**
3. **Data Structuring………………………………………………………………………………………………S3**
4. **Demonstration…………………………………………………………………………………………………S6**
5. **Questions and Feedback………………………………………………………………………………….S9**

1. Getting Started:

You can access the tool through the following URL: https://hannahhapich.shinyapps.io/match/
Or run locally on your device through deploying the application source code available at: https://github.com/hannahhapich/MaTCH

Once you enter the program, you will be taken to the home page:

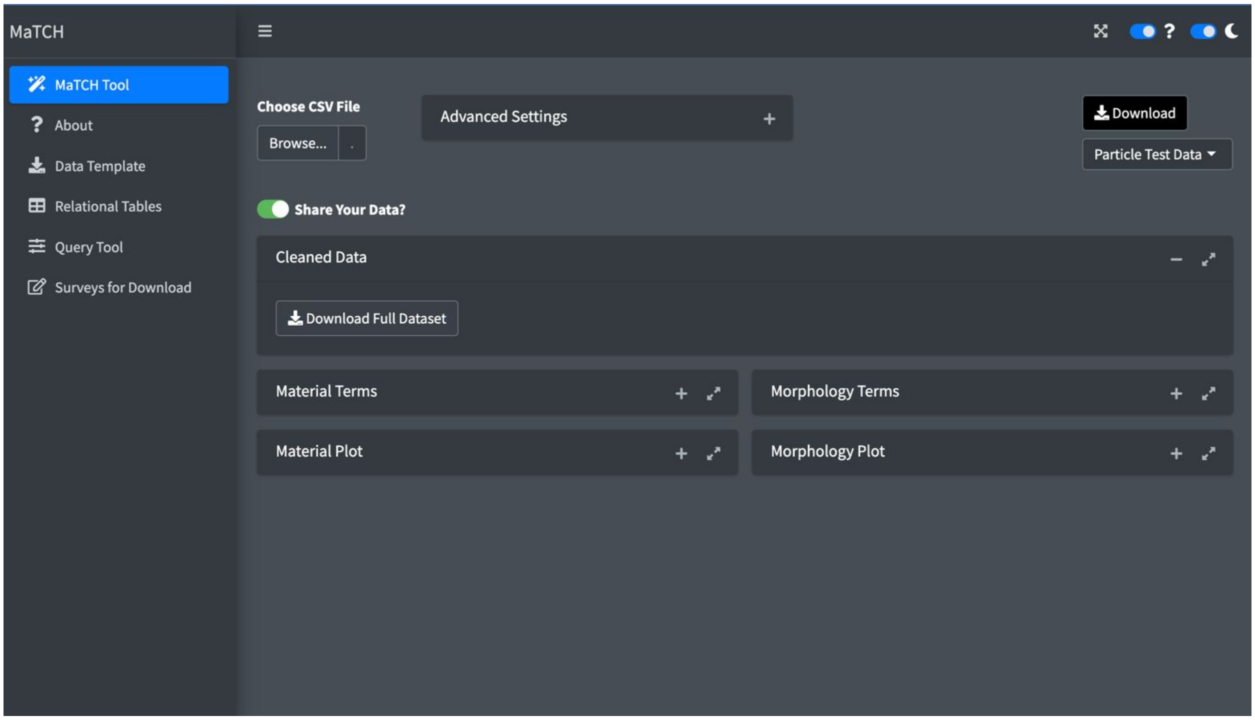

2. Navigation:

On the left, you'll see the navigation pane directing you to the following tabs:

MaTCH Tool: Default home page that allows user uploads to be run through the MaTCH tool.

About: General information about the tool and it's development and acknowledgement of funders.

Data Template: Interactive menu to direct users on how to structure their data, and information on what data cleaning operations can be performed.

Relational Tables: Hosts all relational tables used in the development of this tool for quick view or for download.

Query Tool: Allows users to query the relational tables via an uploaded survey sheet to inform future data structuring.

Surveys for Download: Pre-made data templates for trash and microplastics surveys informed by our study.

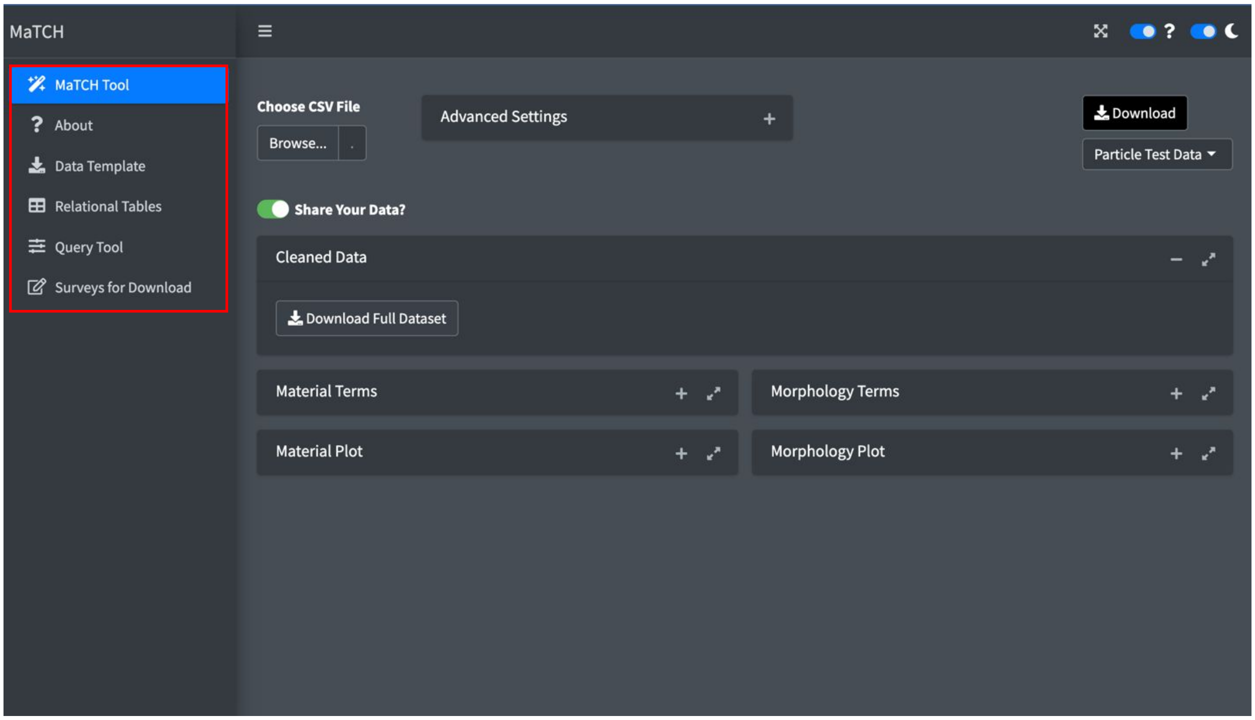

3. Data Structuring:

As MaTCH can process many different data formats, this data structuring tab is provided to guide users through the formatti ng process. It will also inform users what data cleaning operations can be performed with the information provided.

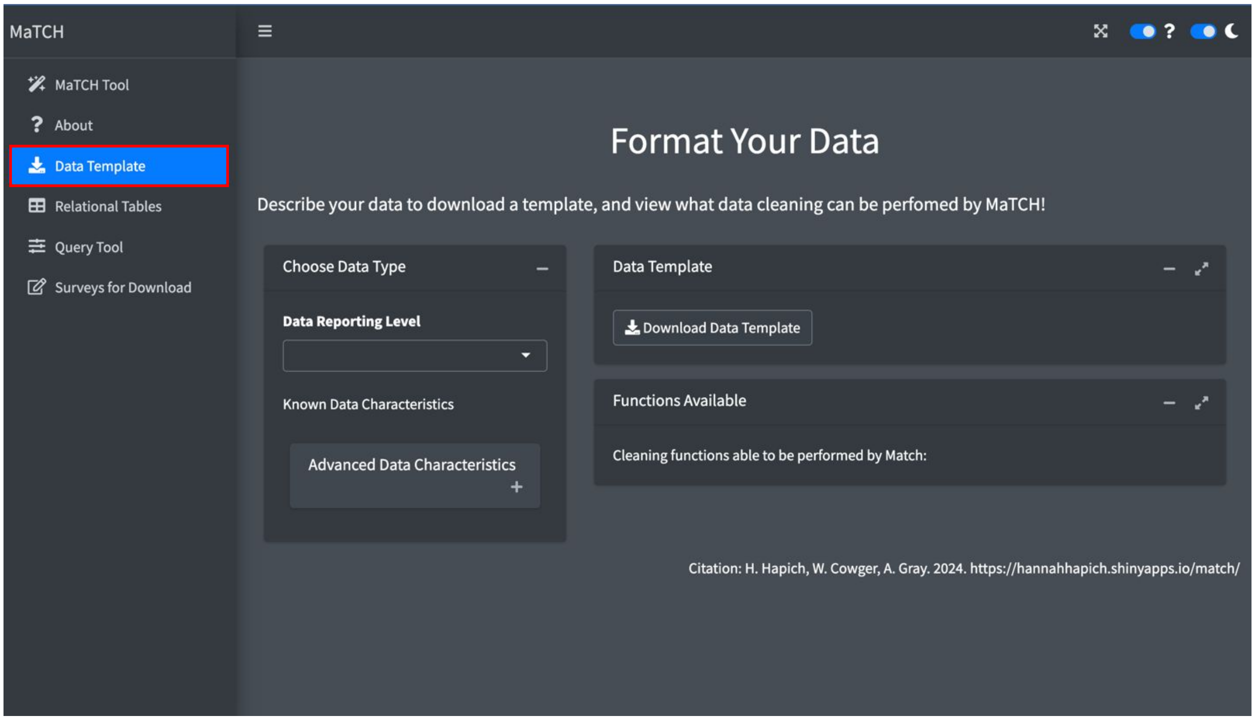

First, users select the data reporting level from the following highlighted dropdown menu. This will either be at the sample level (most common) where concentrations are provided as a particle count, or at the particle level, where each row contains detailed information for each particle found.

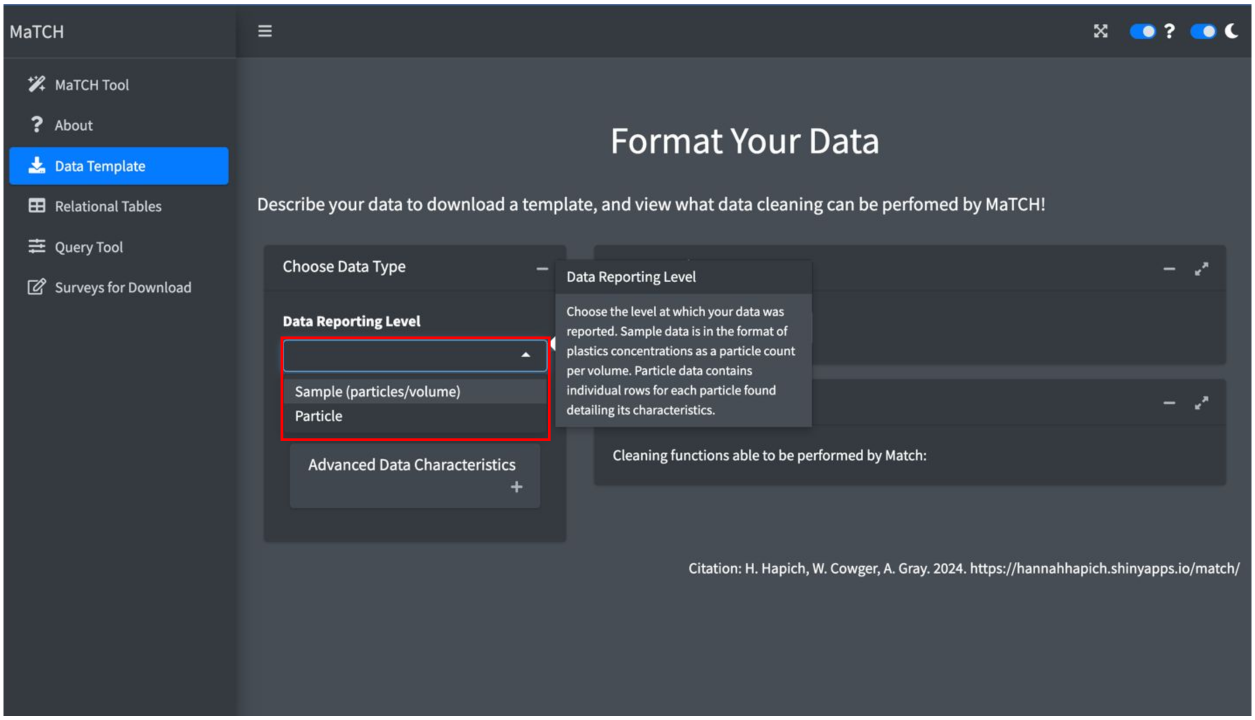

Once a selection is made, the data template with the minimum information required for processing will appear in the upper right, and checkboxes will populate below the dropdown menu on the lower left. These checkboxes describe possible sample or particle characteristic data users may have available that can be used in processing.

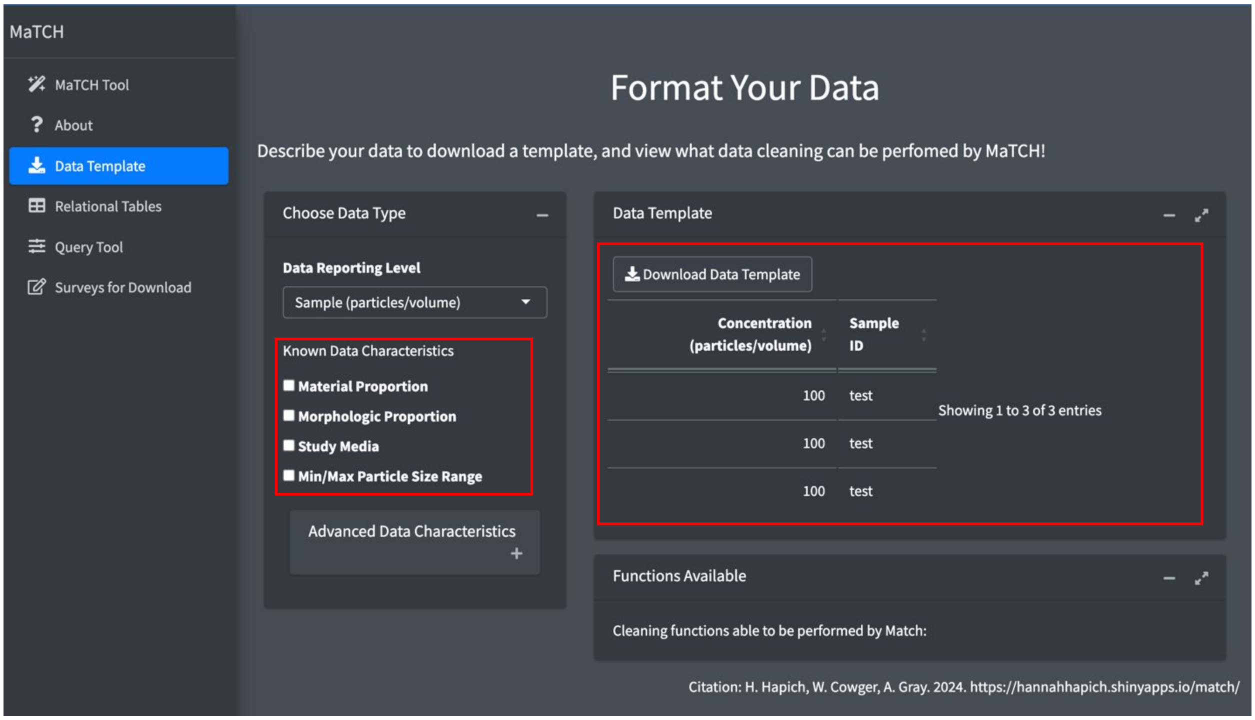

As you select from the “Known Data Characteristics” checkboxes, you will see in the lower right side the data cleaning functions that can be performed with the information provided. Also, see the “Advanced Characteristics” expandable box for additional, less often recorded data characteristics that can be used to enhance accuracy.

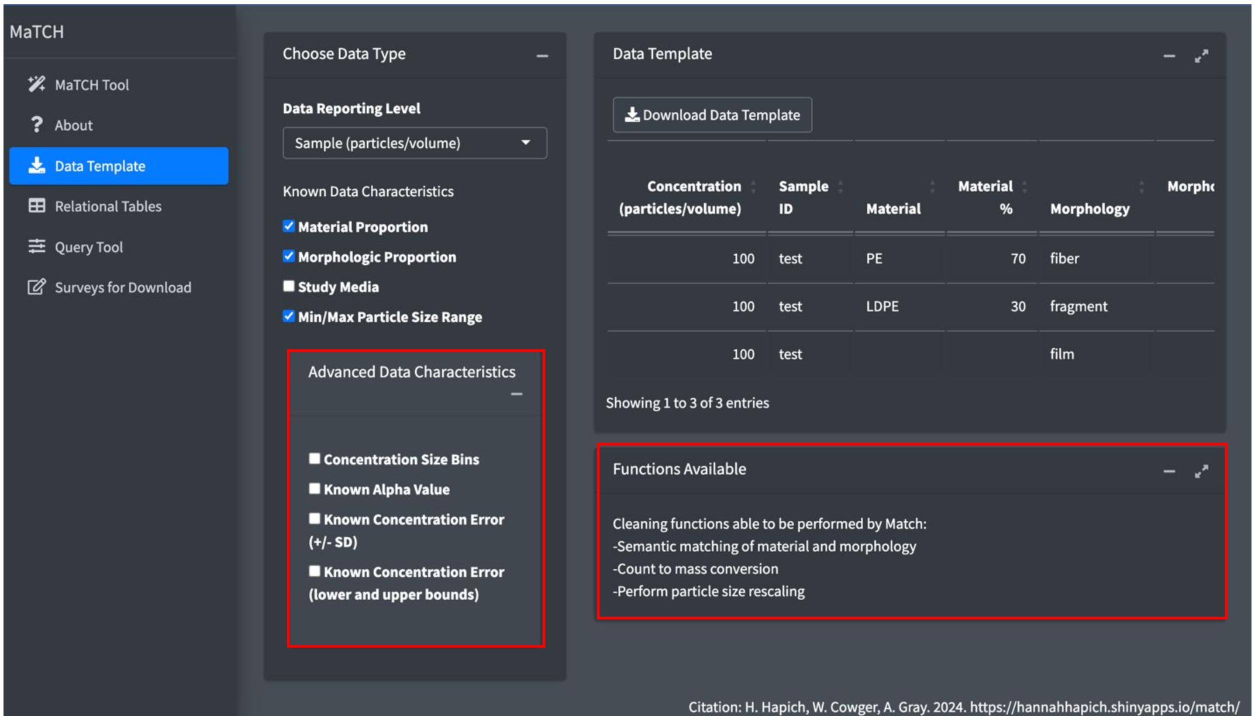

Once you have made your selections, download the data template (which includes three rows of mock data for clarity)

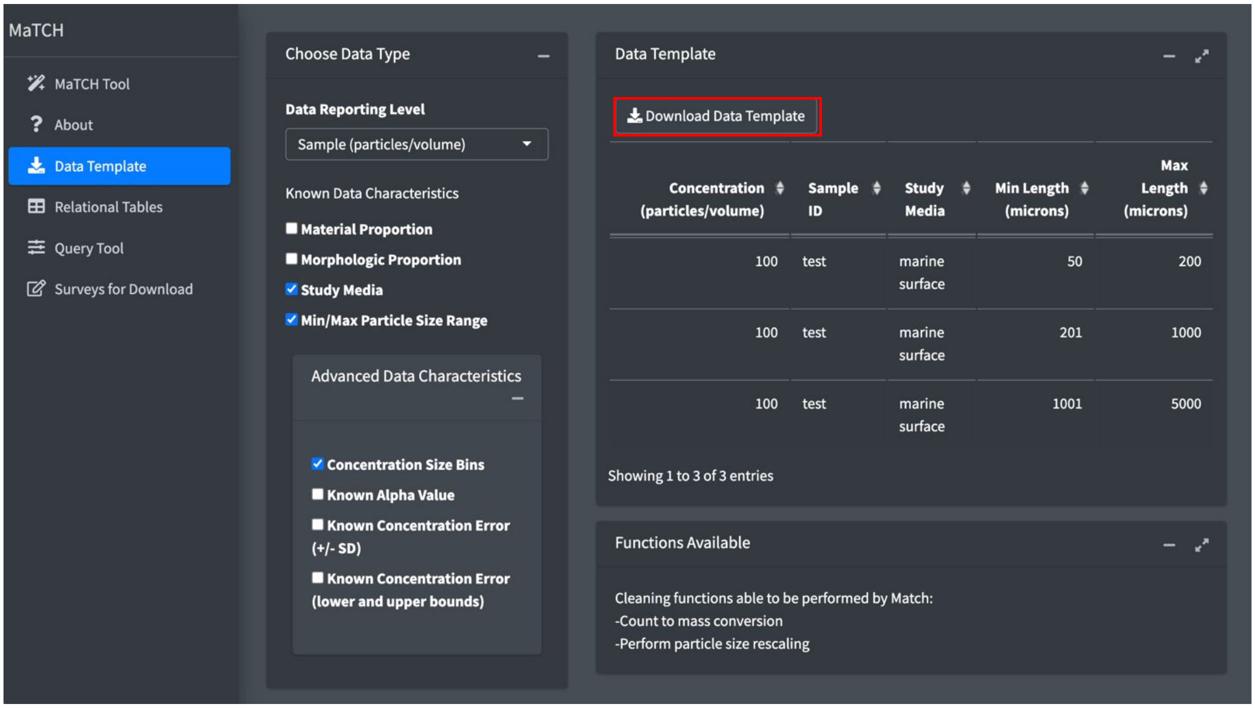

4. Demonstration:

As a practical demonstration, we will show the before and after results using data to reproduce results from this study. See attached in our data repository corresponding to this publication (https://zenodo.org/records/10783982) in the folders Figures > Data and select from any of the following files: “Concentration_Data.csv”, “Cowger_Trash_Data.csv”, or “”Jarlskog_Microparticle_data.csv”. You will also see the corresponding MaTCHed datasets for each of these files (titled “Concentration_Data_Matched.csv”, “Cowger_Trash_Data_Matched.csv”, and “”Jarlskog_Microparticle_data_Matched.csv”, respectively), which will be the output of this demonstration (Note: Results when using the live URL link to this tool are subject to change as the application is updated and methods evolve. See the GitHub repository associated with this publication at https://github.com/hannahhapich/MaTCH/releases/tag/Publication to access the software version being referenced here).

For the purposes of this demonstration, we will be using the file “Concentration_Data.csv”. It will look like the following dataset:

DOI	Sample ID	Study Media	Concentration (particles/volume)	Min Length (microns)	Max Length (microns)
10.1016/j.sciotenv.2018.08.102	Mineral Water.1 | 10.1016/j.sciote	drinkingwater	2649	0.2	5000
10.1016/j.sciotenv.2018.08.102	Mineral Water.2 | 10.1016/j.sciote	drinkingwater	4889	0.2	5000
10.1016/j.sciotenv.2018.08.102	Mineral Water.3 | 10.1016/j.sciote	drinkingwater	6292	0.2	5000
10.1016/j.sciotenv.2018.08.102	WTP1_Treated | 10.1016/j.scioten	drinkingwater	443	0.2	5000
10.1016/j.sciotenv.2018.08.102	WTP2_Treated | 10.1016/j.scioten	drinkingwater	338	0.2	5000
10.1016/j.sciotenv.2018.08.102	WTP3_Treated | 10.1016/j.scioten	drinkingwater	628	0.2	5000
10.1371/journal.pone.0236838	Mintenig | 10.1371/journal.pone.(	drinkingwater	7.00E-04	0.2	5000
10.1371/journal.pone.0236838	Shruti | 10.1371/journal.pone.02:	drinkingwater	18	0.2	5000
10.1371/journal.pone.0236838	Strand | 10.1371/journal.pone.02	drinkingwater	0.59	0.2	5000
10.1371/journal.pone.0236838	Zhang | 10.1371/journal.pone.02:	drinkingwater	0.7	0.2	5000
10.1371/journal.pone.0236838	Mason.1 | 10.1371/journal.pone.(	drinkingwater	315	0.2	5000
10.1371/journal.pone.0236838	Mason.2 | 10.1371/journal.pone.(	drinkingwater	10.4	0.2	5000
10.1371/journal.pone.0236838	Ossmann | 10.1371/journal.pone	drinkingwater	3074	0.2	5000
10.1371/journal.pone.0236838	Weisheu | 10.1371/journal.pone.(	drinkingwater	0.33	0.2	5000
10.1016/j.impact.2021.100302	MineralWater_SingleUsePET | 10.:	drinkingwater	2649	0.2	5000
10.1016/j.impact.2021.100302	MineralWater_ReusablePET | 10.1	drinkingwater	4889	0.2	5000
10.1016/j.impact.2021.100302	MineralWater_Glass | 10.1016/j.ir	drinkingwater	6292	0.2	5000
10.1016/j.impact.2021.100302	RawDrinkingWater.1 | 10.1016/j.ii	drinkingwater	1473	0.2	5000
10.1016/j.impact.2021.100302	RawDrinkingWater.2 | 10.1016/j.ii	drinkingwater	1812	0.2	5000
10.1016/j.impact.2021.100302	RawDrinkingWater.3 | 10.1016/j.ii	drinkingwater	3605	0.2	5000
10.1016/j.impact.2021.100302	TapWater | 10.1016/j.impact.202:	drinkingwater	440	0.2	5000
10.1016/j.impact.2021.100302	RawWater | 10.1016/j.impact.202	drinkingwater	6614	0.2	5000
10.1016/j.impact.2021.100302	TreatedDrinkingWater2 | 10.1016	drinkingwater	930	0.2	5000
10.1016/j.scitotenv.2020.14023	Milence_Fibers.10.1016/j.scitote	drinkingwater	2.8	0.2	5000

From here, navigate to the homepage titled "MaTCH Tool" and simply either drag-and-drop or select the file from its location on your device, and MaTCH will do the rest! Also, notice the processing bar on the lower right corner. Allow around a minute for MaTCH to calculate concentration masses as this process involves simulation of individual particle data for all 1186 rows.

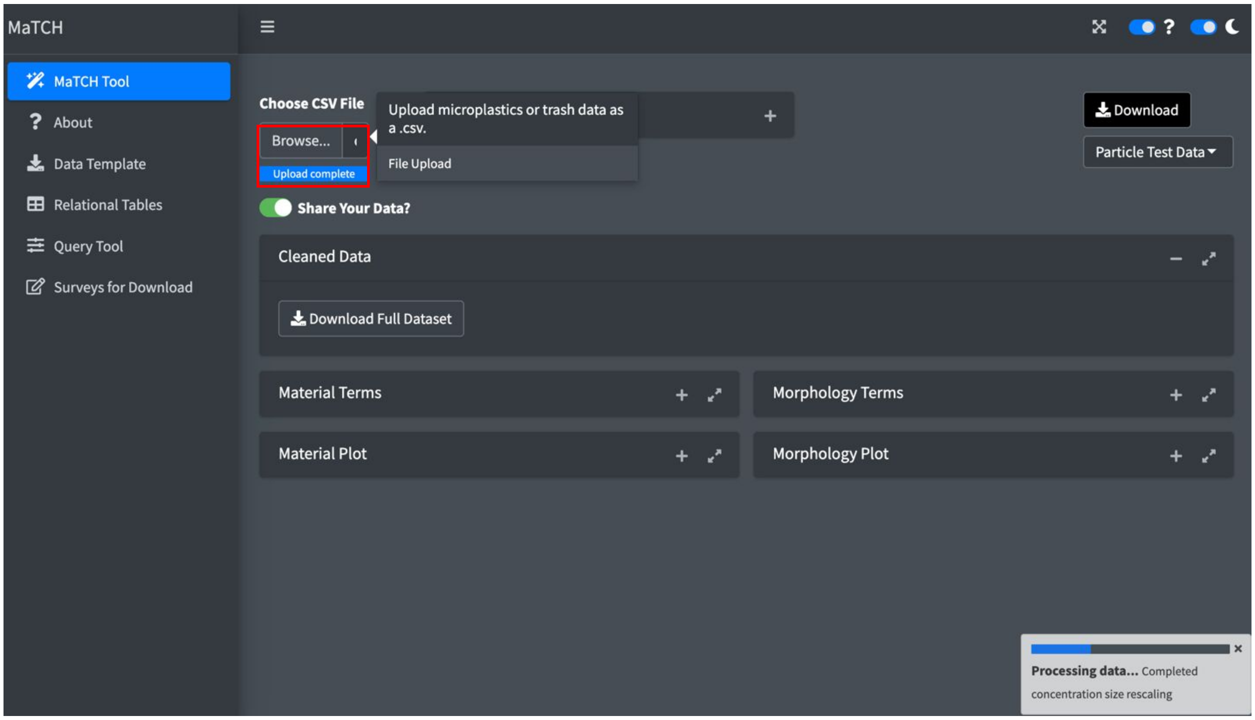

Once processing is complete, scroll to the right to see all corrected concentration values that correspond to values used in this study.

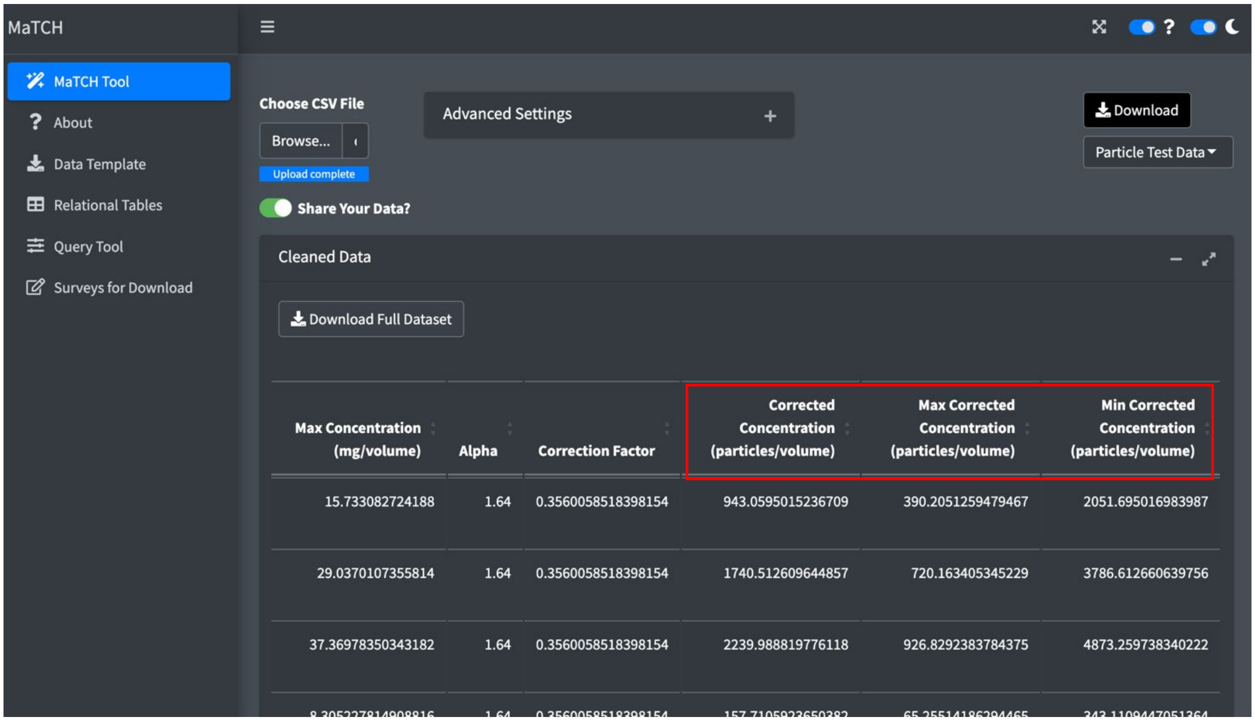

An advanced settings applicable to this use case includes, under “Rescaling Settings,” the adjustment of the corrected size range (default to 1-5000 μm).

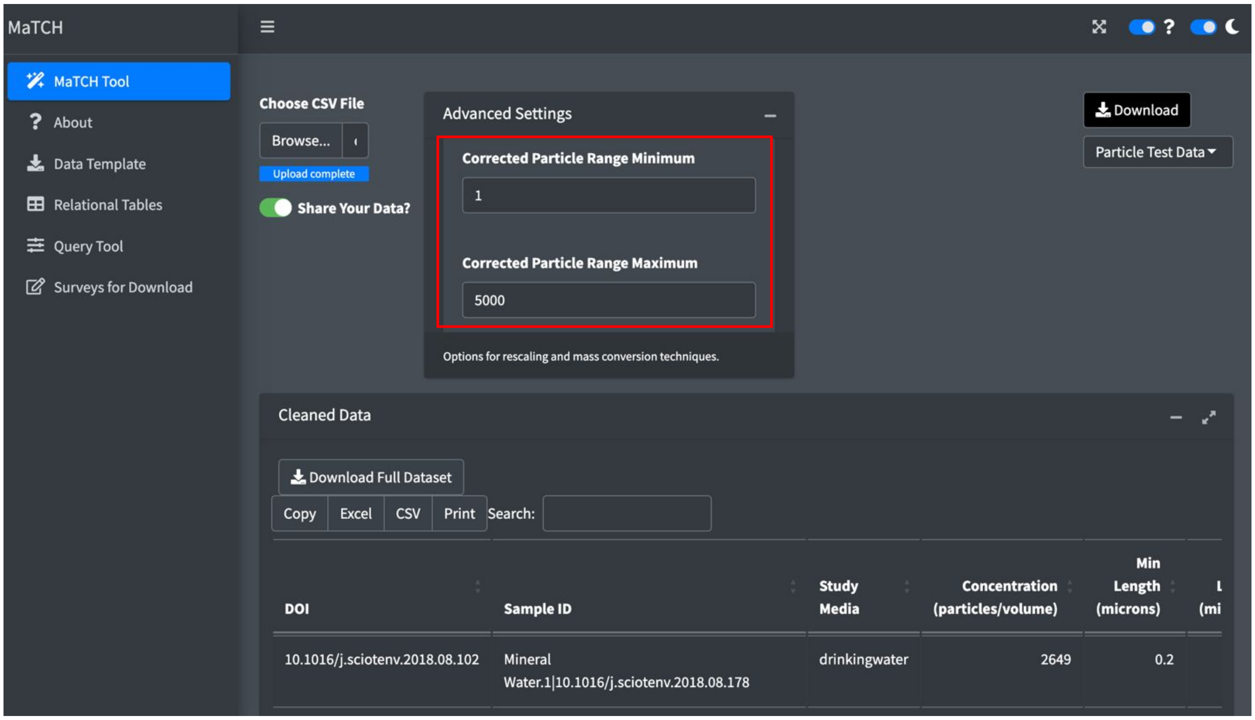

There are plenty of other features to explore, including options for binning techniques when fitting a particle size distribution to individual particle data, allowing for a weighted average of densities to be applied to particles with unknown polymer information, and the generation of sunburst plots similar to those included in this manuscript.

5. Questions and Feedback:

If you have any questions on the usage of this tool, or any suggestions on how we can improve its performance or the user experience, please reach out either via email (hannahhapich@gmail.com) or open an issue/start a discussion on this project's GitHub page (https://github.com/hannahhapich/MaTCH). Our aim is to provide useful, open-source tools for the microplastics and trash research community, so don't hesitate to let us know how we can help grow this tool to keep up with our fast-paced field!
